# Synergistic Effects of PARP Inhibition and Cholesterol Biosynthesis Pathway Modulation

**DOI:** 10.1158/2767-9764.CRC-23-0549

**Published:** 2024-09-16

**Authors:** Anna Rutkowska, H. Christian Eberl, Thilo Werner, Marco L. Hennrich, Daniel C. Sévin, Massimo Petretich, James P. Reddington, Shirin Pocha, Stephan Gade, Amalia Martinez-Segura, Dmytro Dvornikov, Joel Karpiak, Gavain M.A. Sweetman, Christian Fufezan, Birgit Duempelfeld, Florian Braun, Christopher Schofield, Hakan Keles, David Alvarado, Zhuo Wang, Keith H. Jansson, Maria Faelth-Savitski, Edward Curry, Katja Remlinger, Euan A. Stronach, Bin Feng, Geeta Sharma, Kevin Coleman, Paola Grandi, Marcus Bantscheff, Giovanna Bergamini

**Affiliations:** 1 Cellzome, GSK, R&D, Heidelberg, Germany.; 2 Genomic Sciences, R&D, GSK, Heidelberg, Germany.; 3 Medicine Design-Computational Sciences, R&D, GSK, Heidelberg, Germany.; 4 Data Streams and Operations, and Data Science and Data Engineering, R&D, GSK, Heidelberg, Germany.; 5 Centre for Organismal Studies, Heidelberg University, Heidelberg, Germany.; 6 Oncology, Synthetic Lethality Research Unit, R&D, GSK, Heidelberg, Germany.; 7 Chemical Biology Core Facility, EMBL Heidelberg, Heidelberg, Germany.; 8 Biostatistics R&D, GSK, Heidelberg, Germany.; 9 Oncology, Advanced Analytics Experimental Medicine Unit, R&D, GSK, Heidelberg, Germany.

## Abstract

An in-depth multiomic molecular characterization of PARP inhibitors revealed a distinct poly-pharmacology of niraparib (Zejula) mediated by its interaction with lanosterol synthase (LSS), which is not observed with other PARP inhibitors. Niraparib, in a similar way to the LSS inhibitor Ro-48-8071, induced activation of the 24,25-epoxysterol shunt pathway, which is a regulatory signaling branch of the cholesterol biosynthesis pathway. Interestingly, the combination of an LSS inhibitor with a PARP inhibitor that does not bind to LSS, such as olaparib, had an additive effect on killing cancer cells to levels comparable with niraparib as a single agent. In addition, the combination of PARP inhibitors and statins, inhibitors of 3-hydroxy-3-methylglutaryl-coenzyme A reductase, an enzyme catalyzing the rate-limiting step in the mevalonate pathway, had a synergistic effect on tumor cell killing in cell lines and patient-derived ovarian tumor organoids. These observations suggest that concomitant inhibition of the cholesterol biosynthesis pathway and PARP activity might result in stronger efficacy of these inhibitors against tumor types highly dependent on cholesterol metabolism.

**Significance:**

The presented data indicate, to our knowledge, for the first time, the potential benefit of concomitant modulation of cholesterol biosynthesis pathway and PARP inhibition and highlight the need for further investigation to assess its translational relevance.

## Introduction

PARP enzymes are a family of 17 proteins in humans that function in the recognition and repair of DNA breaks, chromatin remodeling, and transcriptional regulation (1, 2). Most enzymes in this family can catalyze the addition of ADP-ribose units to target proteins (PARylation). PARP1 and PARP2 are activated at sites of DNA single-strand breaks (SSB), resulting in the posttranslational modification of various nuclear substrates required for the repair of SSBs.

Synthetic lethality was first described by geneticists to indicate a situation whereby the loss of either gene A or gene B alone has little or no effect on cell viability, but the loss of both genes results in cell death (3). The demonstration that tumor cells containing deleterious mutations in breast cancer (*BRCA*) gene 1 (*BRCA1*) were more sensitive to PARP inhibition than *BRCA* wild-type cells led to the development of PARP inhibitors, leading the FDA to approve PARP inhibitors as the first drugs designed to exploit synthetic lethality for cancer therapy (4). Because auto-PARylation is required for the release of PARP from sites of SSBs, PARP inhibitors prevent the release of the protein from the repaired DNA, a phenomenon called PARP trapping. Trapping PARP proteins on DNA induces stalling of the replication machinery, which produces double-strand breaks, and eventually death of cells with defects in double-strand break repair (5, 6).

Over the last decade, various PARP inhibitors have been involved in more than 70 clinical trials, with the first FDA approval in 2014 for olaparib (Lynparza, AstraZeneca; ref. 7), followed by the approval for rucaparib (Rubraca, Clovis Oncology; ref. 8), niraparib (Zejula, Tesaro/GSK; ref. 9), and talazoparib (Talzenna, Pfizer; ref. 10). Overall, PARP-targeted therapies represent a breakthrough for the treatment of recurrent ovarian cancer (including fallopian tube and primary peritoneal cancer), with two of these inhibitors, olaparib and niraparib, approved for first-line maintenance after platinum chemotherapy (11, 12). In addition, PARP inhibitors are approved for *BRCA*-mutated breast, pancreatic, and prostate cancer, and are under investigation in clinical trials for other tumor types, including lung cancer (13).

One enabling aspect of precision medicine is a deep molecular understanding of drug mechanism of action, which, in combination with the specific background of the patient, can provide a rationale for the choice of a specific compound within the same drug class. Reported differences between approved PARP inhibitors include their PARP-trapping efficiency, potency for tumor cell killing, and other parameters, such as pharmacokinetics, drug–drug interactions, and adverse effects (14).

Here, we report a multiomic characterization of the molecular profile of selected PARP inhibitors. We found that niraparib is highly selective toward PARP1 over other PARP family members and identified lanosterol synthase (LSS) as novel off-target with low micromolar affinity binding. Niraparib, in a similar way to the LSS inhibitor Ro-48-8071, induced activation of the 24,25-epoxysterol shunt pathway, which is a regulatory signaling branch of the cholesterol biosynthesis pathway, pointing out to a distinct poly-pharmacological activity not observed with other PARP inhibitors such as olaparib and talazoparib. Notably, we observed that the triple-negative BRCA (TNBC) cell line HCC70, which is sensitive to modulation of cholesterol homeostasis, undergoes increased cell killing following niraparib treatment than it does after treatment with olaparib. Importantly, the combination of olaparib and this LSS inhibitor, caused a similar degree of cell killing as niraparib monotherapy, suggesting that the concomitant modulation of the cholesterol biosynthesis pathway and PARP signaling could potentially explain niraparib’s higher cytotoxic effect. Finally, combination of either niraparib or olaparib with potent cholesterol biosynthesis pathway inhibitors, such as statins, resulted in synergistic tumor killing in a subset of cell lines and patient-derived cancer organoids. These data suggest that concomitant modulation of cholesterol metabolism might provide additional efficacy to PARP inhibitors and further investigations should assess its translational relevance.

## Materials and Methods

### Commercial chemicals

Niraparib (CAS: 1613220-15-7), talazoparib (CAS: 1207456-01-6), and olaparib (CAS: 763113-22-0) were obtained from the GSK compound library collection; Ro-48-8071 (CAS: 189197-69-1) was obtained from MedChem Express (#HY-18630A); atorvastatin, anhydrous (CAS: 134523-03-8) was from Sigma (#PZ0001-25 mg, anhydrous); pravastatin (CAS: 81131-70-6) was from Sigma (#524403); and fluvastatin (CAS: 93957-55-2) was obtained from Merck/Calbiochem (#344095).

### Chemical synthesis

A detailed description of the synthesis protocol and chemical properties of TCO-PAL-PARP probe (R,E)-cyclooct-4-en-1-yl (2-(3-(3-((5-cyano-6-(4-(3-((4-oxo-3,4-dihydrophthalazin-1-yl)methyl) benzoyl)piperazin-1-yl)pyridin-3-yl)amino)-3-oxopropyl)-3H-diazirin-3-yl)ethyl)carbamate are provided in the “Supplementary Documents” section.

### Human subjects

The human biological samples were ethically sourced, their research was conducted in accord with the recognized ethical guidelines of the Declaration of Helsinki, and approved by the Ethical Committee of the Landesärztekammer Baden-Württemberg, Germany (#2019_CZ_SingleCell) and approved by Institutional Review Board/Independent Ethics Committee. A written informed consent has been obtained from all subjects. Venous blood from consented healthy adult volunteers was sourced ethically at the Heidelberg Blood Centre (IKTZ). The human placenta was supplied by Biopredic International (Saint Grégoire, France).

### Cell lines

All cell lines were accessed from internal GSK collections and originated from ATCC, if not otherwise stated. All cell lines were authenticated using the Promega Cell ID system and the generated short tandem repeat profiles matched the expected short tandem repeat profiles of the ATCC lines. All cell lines were routinely checked for morphology and Mycoplasma contamination (during each process of a stock generation and additionally also in parallel to experiments). The number of passages of reported cell lines between thawing and their application for the described experiments was not exceeding 11. UWB1.289 (RRID:CVCL_B079; female), UWB1.289+BRCA1 (RRID:CVCL_B078); female), K-562 (RRID:CVCL_0004, CCL-243; female), T-47D (RRID:CVCL_0553, GSK ID119699/HTB-133; female), HepG2 Princen (RRID:CVCL_0027, GSK ID97134/HB-8065; male; from Gaubius Laboratory, TNO-PG), MDA-MB-453 (RRID:CVCL_0418, ACC 65; female; from DSMZ), MDA-MB-231 (RRID:CVCL_0062, GSK ID117996/HTB-26; female) is licensed with the University of Texas MD Anderson Cancer Center (UTMDAC), HEK293 (ATCC Cat# CRL-1573, RRID:CVCL_0045; not annotated /human embryo) is licensed with AdVec, the three cell lines: HCC70 (RRID:CVCL_1270, CRL-2315; female), HCC1954 (RRID:CVCL_1259, CRL-2338; female), HCC1806 (RRID:CVCL_1258, CRL-2335; female) are licensed with and originated from University of Texas Southwestern.

Reagents for cultivation were obtained from Gibco unless stated otherwise. Cells were cultured at 37°C, 5% CO_2_ in the following media: UWB1.289: 1:1 RPMI1640:MEGM (Lonza, #CC-3151), 3% FBS, MEGM SingleQuots supplements (Lonza, #CC-4136) used without gentamycin-amphotericin; UWB1.289+BRCA1: as UWB1.289 plus 200-μg/mL G-418 (Geneticin); MDA-MB-231, K-562: RPMI 1640, 1-mmol/L sodium pyruvate, 2-mmol/L Glutamax, 10% FBS; HCC70, HCC1806, and HCC1954: RPMI 1640, 2-mmol/L Glutamax, 5% FBS; T47D: 1:1 DMEM:F12 Ham’s nutrient mix, 2-mmol/L Glutamax, 10% FBS (heat inactivated). MDA-MB-453: RPMI, 10% FBS, 2-mmol/L Glutamax; HEK293: DMEM, 0.1 mmol/L nonessential amino acids, 10% FBS; HepG2 Princen: Basal Medium Eagle, 1-mmol/L sodium pyruvate, 2-mmol/L Glutamax, 0.1-mmol/L nonessential amino acids, and 10% FBS (heat inactivated).

### Ovarian organoid culture

Cryopreserved ovarian tumor organoids were obtained from Hubrecht organoid technology (HUB, Netherlands). Cultures were maintained and expanded in 10 μL droplets of 80% Matrigel (Corning, #356231) in six-well suspension plates (Greiner, #657185) and ovarian cancer (OC) medium as previously described (15), except recombinant R-spondin (R&D Systems, #3500-RS-025/CF) was used at 25-μg/100 mL complete medium. Organoids were passaged 1:2 every 7 days by mechanical shearing using a filtered P1000 pipette tip with a nonfiltered P10 pipette tip on the end. Large and dense organoids were sheared as before but in pre-warmed (37°C) 50% TrypLE (Gibco, #12604013) + 50% ADF+++ (Advanced DMEM with Nutrient Mixture F-12 Ham’s, Gibco, #12634-010), 2-mmol/L Glutamax (Gibco, #35050-038), 10-mmol/L HEPES (Gibco, #15630-056), and 100-U/mL penicillin/streptomycin (Gibco, #15140-122). Sheared organoid pellets were washed with ADF+++, centrifuged at 450 × *g* for 5 minutes, and any residual Matrigel removed before resuspending in fresh 80% Matrigel and re-plating. Plates containing fresh droplets were inverted and incubated at 37°C, 5% CO_2_ in the air for 30 minutes until Matrigel had set, then flipped over gently before adding 2-mL OC medium and incubating as before. OC medium was replaced every 2 to 3 days.

### Cell treatments for omics analysis

Three 15-cm dishes per condition were prepared by seeding 3 to 4 × 10^6^ cells per plate; 24 hours later medium was removed and 25 mL fresh medium containing DMSO (Sigma) or corresponding compound concentration (see figure legends) was applied to the cells. Cells were incubated for the indicated time (24 or 48 hours) at 37°C, 5% CO_2_. Cells were collected by trypsinization, washed twice with PBS, and counted using a Casy Cell Counter (OMNI Life Science). Cell pellets were generated containing 1 to 2 million cells for proteomic and transcriptomic analysis, and 4 to 5 million cells for metabolomic analysis.

### Cell viability assays

Cells were seeded into 96-well plates (2,000–3,000 cells per well depending on the cell line, in 100 μL) and incubated at 5% CO_2_ and 37°C. The day after seeding, 50-μL media was removed and a 50-μL new medium containing either DMSO (Sigma) or corresponding compounds (at double the final assay concentration) was added (0.2% final DMSO concentration). All compounds were serially diluted with a 2-fold or 4-fold dilution factor and assayed over six concentrations of PARP inhibitor and statins or seven concentrations (Ro-48-8071). See individual figures for specific assay concentrations. Experiments were performed with three or four biological replicates, each of which contained three or four technical replicates (technical replicates were on separate 96-well plates with identical layouts, prepared and assayed together). After 7-day incubation with the compound, cell viability was assessed using CellTiter-Glo (Promega) measured on an Envision 2105 plate reader (PerkinElmer) in luminescence mode.

### Ovarian organoid viability assays

Organoids were harvested into a single tube following a 1-hour incubation at 37°C with 1 mg/mL dispase (Gibco, #17105-041) in OC media to dissolve the Matrigel droplets. Organoids were passed through a 70 μm cell strainer (pluriSelect, #43-50070-01), the flow-through collected onto a 20-μm cell strainer (pluriSelect, #43-50020-01) and backwashed into a collection centrifuge tube (organoids 20–70 μm in diameter), which was centrifuged at 450 × *g* for 5 minutes, washed with ADF+++, and centrifuged as before. Organoids were resuspended in a small volume and counted using the CytoSMART counter (Corning). Organoids were seeded into 384-well ultra-low attachment plates (PerkinElmer, #6057302) at 200 organoids per well in OC media plus 5% Matrigel and incubated in a humidified atmosphere of 5% CO_2_ at 37°C for 1 hour before the addition of compounds. For compound treatment, niraparib and atorvastatin were tested at compound-specific starting concentrations (niraparib 30 μmol/L, atorvastatin 5 μmol/L) in a seven-point dose response using a 3-fold dilution factor while keeping the DMSO concentration at 0.5%. Five replicate plates were prepared per condition tested. Compound plates were diluted with OC media comprising 5% Matrigel before addition by Bravo (Agilent) liquid handler. Incubation was performed using MicroClime environmental lids (Labcyte, #LLS-0310) following the manufacturer’s instructions. After a 7-day incubation, cell viability was measured using CellTiter-Glo 3D (Promega). Average luminescence [adenosine triphosphate (ATP) levels] was measured on a PHERAstar (BMG Labtech) plate reader using the MARS application software, and the resulting data were analyzed using TIBCO Spotfire (v12.0) software.

### Affinity enrichment chemoproteomics

Large-scale preparation of cell extracts from frozen cell pellets or tissue was done as described previously (16).

Affinity enrichment chemoproteomics were performed as described previously (16). PARP family-centric bead mix (17) and kinase-centric bead mix [kinobeads (18)] were generated as described before. Niraparib was immobilized via its piperidine nitrogen on N-hydroxy-succinimide (NHS)–Sepharose beads (NHS-activated Sepharose 4 Fast Flow, GE Healthcare Life Sciences) as described before (16). Experiments with PARP-centric beads were done in a combined depletion dose–response setting in MDA-MB-231 lysates (because of the high abundance of proteins from PARP and TNK families; ref. 16) with the first four tandem mass tag (TMT) channels used for determination of the depletion factor and six channels used for competition with either olaparib, talazoparib or niraparib in a six-point dose–response setting with a starting concentration of 30 μmol/L and a dilution factor of 7. Kinobeads experiments were performed in a 1:1:1:1 lysate mix of HEK293, K-562, placenta, and HepG2 Princen (to achieve high coverage of kinases) with competition by either niraparib, talazoparib or olaparib in a nine-point dose–response setting with a starting concentration of 30 μmol/L and a dilution factor of 5. Depletion factors for kinobeads were determined in a separate experiment. Experiments with immobilized niraparib were performed in a combined depletion dose–response setting with the same lysate mix as above, with competition by either niraparib, talazoparib, or olaparib with a six-point dose response with a starting concentration of 30 μmol/L and a dilution factor of 5. pIC50s calculated from the dose–response treatments were corrected for the influence of the bead matrix on the equilibrium to derive apparent dissociation constants pK_d_^app^s as described before (16). All experiments were performed in triplicate, and mean pK_d_^app^s were reported. PARP phylogenetic tree was adapted from ref. 19; kinome tree mapping was performed with the Kinmap tool (20).

### Thermal proteome profiling

Thermal proteome profiling was performed in live UWB1.289 and T47D cells in a two-dimensional thermal proteome profiling (2D-TPP) setting as described before (21, 22). In brief, cells were treated with four different compound concentrations (30, 6, 0.85714, and 0.12245 μmol/L or DMSO control) and incubated at 37°C and 5% CO_2_ for 90 minutes (UWB1.289) or 48 hours (T47D) and then harvested by trypsinization and centrifugation. Cells were resuspended in PBS and transferred to 96-well PCR plates. Cells were heated for 3 minutes to one of the 12 tested temperatures (42.1°C, 44.1°C, 46.2°C, 48.1°C, 50.4°C, 51.9°C, 54°C, 56.1°C, 58.2°C, 60.1°C, 62.4°C, and 63.9°C). Cells were lysed with Igepal CA-630 0.8%, MgCl_2_ 1.5  mmol/L, and benzonase 1 kU ml^−1^, and the aggregated proteins were removed by centrifugation through 0.45 μm filter plates. All flow-throughs from two adjacent temperature treatments were combined into a multiplexed TMT10 experiment. Data analysis was performed as described before (22) and proteins were called significantly affected if they fulfilled the following criteria: stabilization of at least 2-fold in at least one temperature and at least 1.8-fold in an adjacent temperature.

Isothermal dose–response experiments were performed as described before (21). UWB1.289 cells were treated with seven concentrations of niraparib or olaparib starting at 30 μmol/L with a dilution factor of 8 (UWB1.289) or 6.25 μmol/L with a dilution factor of 6 (HCC70) and a DMSO control. The concentration ranges were chosen to optimally cover the potency range for both PARP1 and PARP2. Cells were heated to 48°C to measure PARP1 and PARP2 thermal stabilization. For the UWB1.289 experiment, the eight samples from both treatments were combined into a single 16-plex TMTpro (23) experiment to enable direct comparison to olaparib and niraparib. For HCC70 experiment, the denatured samples were further diluted in the Western Assay System buffer (Protein Simple, #042-195) and relative abundance of PARP1 (Abcam Cat# ab32071, RRID:AB_777100) and superoxide dismutase 1 (SOD1; Novus Cat# NBP1-90186, RRID:AB_11027895) were analyzed by capillary gel electrophoresis (Western Assay System, Protein Simple). The integrated peak areas of PARP1 were divided by the integrated peak areas of SOD1 to correct for potential variation in total protein content. The vehicle condition (DMSO) was used as the reference for fold-change calculations for the different compound concentration conditions. Stabilization was normalized to values between 0 and 1, independent datasets were combined, and a combined dose–response fit was performed to generate pEC_50_s.

### Photo affinity labeling

T47D cells were treated with compound: niraparib (5 μmol/L), olaparib (5 μmol/L), or Ro-48-8071 (2 μmol/L) in phenol red-free culture medium in a 15 cm dish for 1 hour before addition of the TCO-PAL-PARP probe (1 μmol/L) and incubation for 3 hours as above, followed by irradiation at 352 nm in a photo-irradiation chamber on ice (BS-03, UVA, Opsytec, Germany) with a dose of 5 J/cm^2^. Cells were lysed by the addition of 600-μL lysis buffer 1 [50-mmol/L tris/HCl, 150-mmol/L NaCl, 1.5-mmol/L MgCl_2_, 5% glycerol, 1-mmol/L NaVO_4_, 25-m NaF, 0.8% Igepal CA-630, 0.75% SDS, complete protease inhibitor mix (Roche), and 1-μL benzonase nuclease per mL buffer], incubated for 10 minutes at room temperature, followed by 20 minutes at 4°C, and lysates were clarified by centrifugation for 20 minutes at 20,000 × *g*. Lysates were diluted with lysis buffer 2 [50-mmol/L tris/HCl, 150-mmol/L NaCl, 1.5-mmol/L MgCl_2_, 5% glycerol, 1-mmol/L NaVO_4_, 25-mmol/L NaF, 0.4% Igepal CA-630, 0.5% SDS, and complete protease inhibitor mix (Roche)] to adjust concentrations of treatments to be combined into the same TMT experiment. Covalently labeled proteins were enriched by incubation of 1-mL lysate with 35-μL neutravidin beads (High Capacity NeutrAvidin Agarose, Thermo Fisher) for 1 hour at 4°, followed by very stringent washing: 10-mL wash buffer 1 (50-mmol/L tris/HCl, 150-mmol/L NaCl, 1.5-mmol/L MgCl_2_, 5% glycerol, 1-mmol/L NaVO_4_, 25-m NaF, 0.4% Igepal CA-630, 0.5% SDS), 6 mL wash buffer 2 (50-mmol/L tris/HCl, 150-mmol/L NaCl, 1.5-mmol/L MgCl_2_, 5% glycerol, 1-mmol/L NaVO_4_, 25-mmol/L NaF, 0.2% Igepal CA-630, and 0.5% SDS), 6-mL digest wash buffer 1 (50-mmol/L HEPES pH 8.5, 400-mmol/L NaCl, and 0.5% SDS), 16-mL digest wash buffer 2 (50 mmol/L HEPES pH 8.5, and 400 mmol/L NaCl), 16-mL digest wash buffer 3 (50-mmol/L HEPES pH 8.5 and 2-mol/L urea), and 6-mL digest wash buffer 4 (50-mmol/L HEPES pH 8.5). Enriched proteins were eluted by on-bead digestion: 60-μL digestion buffer [50-mmol/L HEPES pH 8.5, 5-mmol/L tris(2-carboxyethyl)phosphine hydrochloride (TCEP), 15-mmol/L chloroacetamide, 0.004-μg LysC/μL, and 0.004 μg trypsin was added to the beads and beads were incubated overnight at room temperature. Eluted peptides were lyophilized, TMT labeled, and analyzed *via* mass spectrometry (MS).

### MS analysis

All proteomic experiments utilized TMT for relative quantification. Measurements and analyses were performed as described before (23). Affinity enrichments and photoaffinity labeling samples were measured without offline fractionation; TPPs and expression proteomics samples were offline fractionated into 24 fractions, of which 8 to 24 fractions were measured. Samples were measured on Therma Orbitrap instruments (Orbitrap Lumos, Q Exactive HF, Q Exactive HFX, or Exploris).

### Differential scanning fluorometry

Recombinant LSS was expressed and purified as described before (24), with some modifications. SF9 cells were infected with recombinant P1 baculovirus encoding 6His-tev-human lanosterol synthase, titred at 8.1 × 10^8^ pfu/mL equal to a multiplicity of infection of 12. The cell pellet from a 1-L baculovirus expression was resuspended to a final volume of 4.44-mL/g buffer A [50-mmol/L tris pH 7.5, 500-mmol/L NaCl, 20-mmol/L imidazole, 2-mmol/L TCEP, and 0.2% (v/v) Triton X-100] containing 1-mL/mL protease inhibitor cocktail (Sigma P8340). The cell suspension was lysed by Dounce homogenization and clarified by centrifugation at 100,000 *g* for 60 minutes at 4°C. The lysate supernatant was applied to a 5-mL HisTrap FF crude column (GE Healthcare 17-5286-01) equilibrated with buffer B [50-mmol/L tris pH 7.5, 500-mmol/L NaCl, 2-mmol/L TCEP, 0.2% (v/v) Triton X-100, and 20-mmol/L imidazole]. The column was washed with buffer B over 10 column volumes and buffer C [50-mmol/L tris pH 7.5, 500-mmol/L NaCl, 2-mmol/L TCEP, 0.2% (v/v) Triton X-100, and 50-mmol/L imidazole] over 10 column volumes. Bound LSS was eluted with a linear gradient of 50- to 250-mmol/L imidazole, over 10 column volumes. Fractions containing LSS were concentrated to 10 mL and further purified by Superdex 200 prep grade column equilibrated with buffer D (50-mmol/L tris pH 7.5, 150-mmol/L NaCl, 2-mmol/L TCEP, and 0.2% [v/v] Triton X-100).

Recombinant LSS (0.75 mg/mL) was incubated with 10-μmol/L niraparib, olaparib, or Ro-48-8071 or DMSO control, and thermal stability was measured on a Prometheus nt.48 (NanoTemper). Melting point was determined as a maximum of d(330 nm/350 nm)dT.

### Modeling LSS compound docking

The crystal structure of an inhibitor bound to lanosterol synthase (PDB ID: 1W6J) was used to model the binding poses of olaparib and niraparib to LSS. Computational modeling was performed using the Schrödinger suite of software (version 2021.01, Schrödinger, LLC, New York). Protein structures were prepared with the Protein Preparation Wizard using default parameters and rebuilding missing side chains using Prime. Hydrogens were added, and hydrogen bond orientations were sampled and optimized using a restrained minimization with the OPLS3e forcefield. The LSS inhibitor Ro-48-8071 was used for Glide receptor docking grid generation. Olaparib and niraparib were prepared for docking using default parameters in LigPrep and Glide XP while enforcing planarity of conjugated pi groups. Post-docking minimization with strain correction terms was used to rank the final output poses. All docked figures were generated with PyMOL (Schrödinger, LLC, New York).

### Flow cytometry analysis of pSTAT5 levels in IL2 stimulated PBMC

Fresh blood was received from a local blood bank (IKTZ Heidelberg). PBMCs were isolated using EasySep Direct Human PBMC isolation kit (StemCell #19654) according to the manufacturer’s instructions; 500,000 cells were plated in 450-μL medium (RPMI with 10% FBS, 1× pen/strep and 1× fungizone, all from Gibco) in 96-well V-bottom deep-well plates (Greiner Bio-One, #786261). Plates were placed overnight into the humidified incubator at 37°C, 5% CO_2_. The next day, cells were pre-treated for 45 minutes with indicated concentrations of DMSO, niraparib, olaparib, or specific Janus kinase 1 (JAK1) inhibitor itacitinib (Selleckchem, CAS No. 1334298-90-6). Cells were stimulated with human recombinant IL2 (PeproTech, #200-02) at a concentration of 500 ng/mL for 15 minutes. PBMCs were then fixed with 1.5% formaldehyde (Fischer Scientific #10751395) for 10 minutes at room temperature, and centrifuged at 5 minutes, 500 × *g*, at room temperature. The cell pellet was resuspended in 300 μL of cold EasySep buffer (StemCell, #20144). Cells were permeabilized by slowly adding 500 μL cold BD Phosflow Perm Buffer III (BD #558050) followed by 30 minutes of incubation at −20°C. Cells were centrifuged 400 *g* for 5 minutes, washed twice with 750 μL of EasySep buffer, and resuspended in 50 μL of master mix containing CD3 APC-R700 1:200 [BD Biosciences Cat# 565119 (also 565120), RRID:AB_2744385], CD4-BUV395 1:100 (BD Biosciences Cat# 564724, RRID:AB_2738917), CD8-BB515 1:200 [BD Biosciences Cat# 564526 (also 564527), RRID:AB_2744458], CD14-BV711 1:400 [BD Biosciences Cat# 563372 (also 563373), RRID:AB_2744290], CD15-BV605 1:800 [BD Biosciences Cat# 562979 (also 562980), RRID:AB_2744292], CD19-BV480 1:200 (BD Biosciences Cat# 566103, RRID:AB_2739505), CD16-BV421 1:1,000 (BD Biosciences Cat# 562878, RRID:AB_2737861), CD45-PE-Cy5 1:200 (BD Biosciences Cat# 560974, RRID:AB_10562039), CD56-PE-CF594 1:800 (BD Biosciences Cat# 564849, RRID:AB_2738983), pSTAT5-PE 1:50 (BD Biosciences Cat# 562077, RRID:AB_10894188), HLA-DR-BV786 1:200 (BD Biosciences Cat# 564041, RRID:AB_2738559), and CD11c-APC 1:400 (BD Biosciences Cat# 559877, RRID:AB_398680) in Brilliant Stain Buffer (BD #563716). After 30 minutes of incubation at room temperature, 180 μL of EasySep buffer was added, and the plate was centrifuged at 400 *g*, for 5 minutes, at room temperature. The cell pellets were resuspended in 150-μL EasySep buffer and transferred into a 96-well U-bottom plate (Becton Dickinson GmbH # 353077) for high throughput sampler acquisition on BD LSRFortessa with FACSDiva 8.0.2 software. The analysis was done with FlowJo V10.7.1 software.

## Steroidomics

### Steroid extraction

The cell pellets were suspended in ethyl acetate, and water was added to create an 80:20 v/v ethyl acetate:water mixture. Picolinic-D_4_ acid (Sigma #615757) labeled steroids were added to each sample as internal standards [steroids esterified with picolinic-D_4_ acid were 24(S),25-epoxycholesterol #700039P-1MG, 24,25-dihydrolanosterol #700067P-5MG, 5α-7,24-cholestadiene #700114P-1MG, 5α-dihydrotestosterone #D-073-1ML, 7-dehydrocholesterol #30800-5G-F, 7-dehydrodesmosterol #700138P-1MG, cholesterol #C8667-1G, cortisol #C-106-1ML, dehydroepiandrosterone #D-063-1ML, desmosterol #700060P-5MG, dihydro-ff-MAS #700177P-1MG, dihydro-T-MAS #700173P-1MG, estradiol 17beta #E8875-1G, estriol #46565-100MG, estrone #E9750-5G, follicular fluid meiosis-activating sterol (FF-MAS) #700077P-1MG, lanosterol #700063P-1MG, lathosterol #700069P-5MG, pregnenolone #700142P-50MG, testosterone #T1500-1G, T-MAS #700073P-1MG, vitaminD3 #C9756-1G, zymostenol #700118P-1MG, and zymosterol #700068P-1MG]. The samples were shaken vigorously followed by centrifugation. The lower aqueous phase was frozen on dry ice, and the upper ethyl acetate phase was transferred to a new vial. The aqueous phase was washed with the same volume of fresh ethyl acetate, and the extraction was repeated. The second extract was united with the first and dried in a vacuum centrifuge. The dry steroids were derivatized with picolinic acid.

### Shiina esterification of steroids with picolinic acid under basic conditions

The reaction mixture contains 25-mg/mL picolinic acid, 10-mg/mL 4-dimethylaminopyridine, and 20-mg/mL 2-methyl-6-nitrobenzoic anhydride in tetrahydrofuran. Internal standards were created using picolinic-D_4_ acid instead of picolinic acid. The reaction mixture was created by adding the 4-dimethylaminopyridine solution to the saturated picolinic acid solution. Subsequently, the 2-methyl-6-nitrobenzoic anhydride solution was added to the turbid mixture. The reaction mixture clears and a precipitate forms, which is not transferred to the dried steroids. The dried steroids were dissolved and derivatized in a 100-μL reaction mixture plus 20-μL triethylamine. After 30 to 60 minutes, the reaction was stopped with 1 mL of 1% acetic acid. The samples were desalted on a C18 solid phase extraction plate, and the eluate was dried in a vacuum centrifuge. The picolinic acid-labeled steroids were reconstituted with methanol and analyzed *via* LC-MS.

### LC-MS analysis

The picolinic acid-labeled steroids were analyzed on an UltiMate LC system (Thermo Scientific) consisting of a WPS-3000TRS autosampler, a SRD-3600 degasser, and an HPG-3400RS ultra-high pressure LC pump coupled to a Q Exactive Plus (Thermo Scientific) mass spectrometer via a heated electrospray ion source HESI-II (Thermo Scientific). The labeled steroids were separated on two Phenomenex Luna C8 2.0 × 150 mm, 3 μm columns connected in series to increase the separation power. Mobile phase A was 0.2% formic acid in water, and mobile phase B was 0.2% formic acid in acetonitrile:isopropanol 50:50 (v/v). The flow rate was set to 0.15 mL/minute. An initial gradient from 50% B to 85% B in 3.5 minutes and isocratic separation at 85% B for 29.4 minutes was followed by a 1-minute gradient from 85% B to 100% B. Subsequently, the columns were washed for 3 minutes with 100% B and equilibrated at 50% B.

All spectra were acquired in positive ion mode. Full scan spectra were recorded in profile mode in a mass range of 180 to 650 m/z, at a resolution of 35,000, with a maximum ion fill time of 100 ms and an automatic gain control (AGC) target value of 3 × 10^6^ ions. A Top2 method was applied with the normalized collision energy set to 30, an isolation window of 1.0, a resolution at 17,500, a maximum ion fill time of 50 ms, and an AGC target value of 1 × 10^5^ ions. The fragmentation spectra were recorded in profile mode. The Top2 method was followed by a parallel reaction monitoring method with the normalized collision energy set to 20, an isolation window of 1.0, a resolution at 17,500, a loop count of 4, a maximum ion fill time of 50 ms, and an AGC target value of 2 × 10^5^ ions. The inclusion list contained the masses of the steroids of interest and their corresponding deuterium-labeled internal standards.

### Steroidomics data analysis pipeline

The raw data files were first converted to mzML files using msconvert [ProteoWizard version 3.0.20287 (25)] before being read with pymzML (26). MS1 quantification was performed with pyQms (27) using the isotopic cluster data for each of the metabolites and their labeled counterparts.

The results of the pyQms quantification were concatenated and analyzed by an in-house solution written in Python (version 3.6 http://www.python.org). First, the quantification results were normalized to any internal standards. Data from calibration samples were collated and calibration lines were fitted using lmfit (28).

Calibration lines were validated by calculating the concentration of each sample from its measured quantification. To be valid the calculated concentration of all data points in the calibration line must be within 15% of the actual concentration. For noncompliant data, an automated outlier removal was applied, in which an increasing number of data points were removed, up to a maximum of 25% of the total number of data points. New calibration lines were calculated and validated using this reduced number of data points. The valid calibration with the greatest number of data points and the highest *R*^2^ value was chosen as the calibration to use for the sample data.

To ensure the best calibration for the data, the calibration procedure was repeated with several linear models. Typically, straight lines (*y* = *ax* + *b*) and quadratic lines (*y* = *ax*^2^ + *bx* + *c*) were fitted, both using unweighted data and also weighting the data by the inverse of the concentration. The best-fitting line as judged by the highest *R*^2^ value was selected as the calibration of the metabolite. The calibration data were then used to calculate the metabolite concentration in each of the samples.

### Transcriptomics

T47D cells (750,000) were suspended in 350-μL RLT buffer (Qiagen) on wet ice, transferred to a QIAshredder tube (Qiagen), and centrifuged at 21,000 × *g* for 2 minutes. RNA was extracted using the RNeasy 96-well plate kit (Qiagen, #74181) according to the manufacturer’s instructions including the DNase step. RNA concentration and integrity were assessed using a fragment Analyzer (Agilent). Libraries were prepared using the NEBNext Ultra II Kit stranded (NEB, #7760) and NEBNext Poly(A) mRNA Magnetic Isolation Module (NEB, #E7490) following the manufacturer’s specifications using the following options: 250 ng of total RNA per sample (starting material), poly(A) enrichment (mRNA isolation), 12 PCR cycles, 10-minute fragmentation time. DNA concentration and library size distribution were determined on a fragment analyzer. Libraries were pooled to 2 nmol/L and sequenced on a NextSeq2000 following the manufacturer’s specifications. FastQ files were generated using the software bcl2fastq (version 2.20).

## Quantification and Statistical Analysis

### Bliss score of synergy calculation

To assess synergy, the Bliss Score was calculated for each plate (entire concentration matrix) using the obtained data (% of viability or % of inhibition) and the SynergyFinder Plus (29) applying whole matrix correction. Heatmaps and 3D synergy maps were generated highlighting the synergistic and antagonistic dose regions in red and green colors, respectively. *P* values and 95% confidence intervals are reported. Synergy was classified as significant when the Bliss Score for the entire concentration matrix was >3 with a *P* value of <0.05, 95% confidence interval was positive, and synergy was detected in one region covering at least two neighboring concentrations of each compound tested.

### Statistical analysis

All cell viability assays were done as indicated in the figure legends (at least two independent biological replicates) and the data are presented as mean ± SEM. For steroidomic experiments mean of technical replicates is shown from an exemplary experiment, *P* values were calculated using a two-tailed unpaired *T* test with Welch’s correction *versus* DMSO. Affinity enrichment chemoproteomics experiments were performed in triplicate, and mean pK_d_^app^s were reported. All data fitting and statistical analysis were performed using GraphPad Prism software (Version 9.2.0), if not otherwise stated.

#### Statistical analysis of transcriptomics data

Raw FastQ files were processed to count matrices using the cloud processing tool DNAnexus. Adaptor trimming was carried out using Trimmomatic (30). Reads were then mapped to the reference human genome (GRCh38.96) with STAR v. 1.3.4 (31). Picard MarkDuplicates v. 2.1.1 tool was used to identify and quantify PCR duplicates (http://broadinstitute.github.io/picard). Reads were assigned to genes using the command featureCounts from the software Subread (32) to produce count matrices. Genes were prefiltered before differential expression testing to include only genes with more than 10 counts total across all samples. Statistics of differential gene expression were calculated with DESeq2 (33). The resulting *P* values were adjusted for multiple testing using the method of Benjamini and Hochberg (34). The criteria to consider a gene differentially regulated was an adjusted *P* value lower than 0.05 and an absolute log_2_ fold change in expression greater than 1.5.

#### Statistical analysis of expression proteomics data

Statistical analysis and visualization of the data were performed using the statistical language R. Proteomics data were filtered for proteins with at least two unique quantified peptides. The log_2_ sum of ion intensities was used as a measure for protein abundance. These abundance measures were normalized using quantile normalization. Differential analysis was carried out using a moderated *t* test implemented in the limma package (35). The resulting *P* values were adjusted for multiple testing using the method of Benjamini and Hochberg (34). A protein was considered statistically significantly different with an adjusted *P* value below 0.05 and log_2_ fold change above log_2_(1.5) or below −log_2_(1.5).

### Visualization of cholesterol biosynthesis pathway

A selected list of genes covering the cholesterol biosynthesis pathway has been visualized on the generated transcriptomics and proteomics data sets: (i) mevalonate and prenylation pathway: ACAT1, ACAT2, SERPINA3, hydroxymethylglutaryl-CoA synthase (HMGCS1), HMGCS2, 3-hydroxy-3-methylglutaryl-coenzyme A reductase (HMGCR), MVK, PMVK, diphosphomevalonate decarboxylase, FDPS, IDI1, IDI2, squalene synthase (FDFT1), squalene monooxygenase (SQLE), HMGCL, FDFT1, GGPS1, FNTB, FNTA, PGGT1B, RABGGTA, RABGGTB, PDSS1, PDSS2, RCE1, ICMT; (ii) cholesterol synthesis: LSS, DHCR7, CYP51A1, TM7SF2, methylsterol monooxygenase 1, NSDHL, HSD17B7, DHCR24, SC5D, EBP, SREBF1, SREBF2, INSIG1, INSIG2, LBR, NR1H2, NR1H3, SOAT1, SOAT2, SCAP, CYP27A1, CYP46A1, CH25H, LIPA; (iii) liver X receptor (LXR) pathways: effux and lipid metabolism: ATP-binding cassette subfamily A member 1, ABCB1, ABCG1, MYLIP, ABCG5, ABCG8, ATP-binding cassette subfamily G member 4, ARL4C, APOE, FASN, stearoyl-coenzyme A desaturase (SCD), SCD1c, MLXIPL, PLTP, SREBP1c, APOC1, APOC2, APOC4, APOD, LPCAT3, and LPL.

### Data availability

All data reported in this manuscript will be shared by the corresponding author upon request. Most of the data generated in this study are available within the article and its supplementary tables. The MS proteomics raw data generated in this study are publicly available in the ProteomeXchange Consortium via the Proteomics Identification Database at PXD044329, PXD044332, PXD045297, PXD044330, PXD044334, PXD044331, and PXD044333. The RNAseq raw data generated in this study are publicly available in Gene Expression Omnibus at GSE230634. This manuscript does not report original codes. Any additional information required to reanalyze the data reported in this manuscript is available from the corresponding author upon request.

## Results

### Comparative profiling of PARP inhibitors

To gain insights into the selectivity and potential poly-pharmacology of the marketed PARP inhibitors olaparib, niraparib, and talazoparib, we performed a comprehensive chemoproteomics profiling by an unprecedented combination of lysate-based and cell-based methodologies to achieve optimal coverage across the proteome. In lysate-based affinity enrichment methods, a bead matrix with one or multiple immobilized small molecules was used to specifically enrich target proteins from human cellular lysates. Co-incubation with a compound of interest over a range of concentrations enables the determination of half maximal inhibitory concentrations (IC_50_) and apparent dissociation constants [pK_d_^app^, −log_10_(K_d_^app^ [M])].

**Figure 1:**
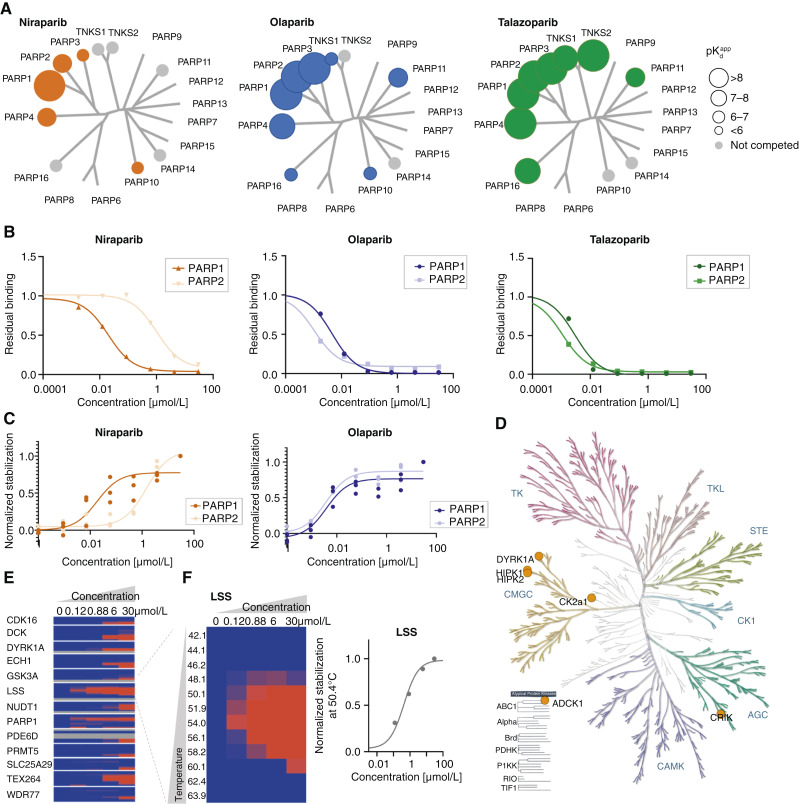
Comprehensive selectivity profile of PARP inhibitors. **A,** Selectivity profiling of talazoparib, olaparib, and niraparib on a PARP selective bead matrix using affinity enrichment chemoproteomics from MDA-MB-231 lysate. Identified targets with their respective mean (*n* = 3) apparent dissociation constants pK_d_^app^ are visualized on a phylogenetic tree of the PARP family (details and statistical analysis in Supplementary Table S1). **B,** Representative dose–response curves for PARP1 and PARP2 derived from **A**. **C,** Quantitative target engagement of PARP1 and PARP2 by olaparib and niraparib (30, 3.75, 0.468, 0.058, 0.007, 0.0009, and 00001 μmol/L) determined by thermal proteome profiling in UWB1.289 cells at 48°C (*n* = 3, details and statistical analysis in Supplementary Table S2). **D,** Selectivity profiling of talazoparib, olaparib, and niraparib on a kinase selective bead matrix from a lysate mix. Identified targets (*n* = 2, details in Supplementary Table S3) for niraparib are visualized on a phylogenetic tree of the kinase family. No kinases were identified for olaparib and talazoparib under the chosen conditions. **E,** Two-dimensional thermal proteome profiling (2D-TPP) in UWB1.289 cells to identify targets and off-targets of niraparib (*n* = 1). Heat maps display relative stabilization compared with the control treatment. Dependencies on compound concentration (*x*-axis: 30, 6, 0.85714, and 0.12245 μmol/L) and temperature (*y*-axis: 42°C–63.9°C) are visualized. **F,** Heatmap visualization (enlarged from **E**) and dose–response curve (at 50.4°C) of thermal stabilization of lanosterol synthase (LSS).

According to previous reports from biochemical assays using recombinant PARP proteins (36–39), the four PARP inhibitors currently approved for clinical use, olaparib, niraparib, talazoparib, and rucaparib, have all similar affinity for both PARP1 and PARP2. By using our target-centric bead matrix to determine selectivity within the PARP family (17), we indeed could confirm that both olaparib and talazoparib bind to PARP1 and PARP2 with similar affinity (pK_d_^app^ PARP1/PARP2: 9.0/>9.1 and 9.2/9.1, respectively, Fig. 1A and B; Supplementary Table S1) and engages further PARP family members such as PARP3, PARP4, and PARP11, including tankyrases (TNKS and TNKS1). In contrast, niraparib’s main target is PARP1 (pK_d_^app^ PARP1: 8.5) with a significant selectivity window to PARP2 (pK_d_^app^ PARP2: 6.2; Fig. 1A and B) as well as to all other PARP family members.

To quantitatively determine target engagement for PARP1 and PARP2 in live cells, we performed an isothermal dose–response experiment (22) and could confirm equal affinity for olaparib toward PARP1 and PARP2 (pEC_50_ PARP1/PARP2: 8.4), whereas for niraparib an 80-fold selectivity window was observed between these two PARP proteins (pEC_50_ PARP1: 7.7, pEC_50_ PARP2: 5.8; Fig. 1C; Supplementary Table S2). Full occupancy of PARP2 by niraparib, and thus, full inhibition of PARP2 enzymatic activity is likely only achieved at high micromolar drug concentrations.

As kinases have been described as niraparib off-targets (38), we next performed selectivity profiling of all three PARP inhibitors with kinobeads, a set of immobilized broad-spectrum kinase inhibitors (Fig. 1D; Supplementary Table S3; ref. 18). Niraparib showed affinity to multiple kinases, mainly within the CMGC [cyclin-dependent kinases (CDK), mitogen-activated protein kinases, glycogen synthase kinases, and CDK-like kinases] family including binding to dual-specificity tyrosine phosphorylation-regulated kinase 1A (DYRK1), whereas olaparib and talazoparib did not show off-targets in this protein family. Notably, all kinase off-targets identified showed pK_d_^app^s above 10 μmol/L.

To explore additional off-targets beyond those covered by the PARP matrix and the kinobeads, we immobilized niraparib via its piperidine nitrogen directly on NHS beads for affinity enrichment and performed competition experiments (Supplementary Fig. S1A and S1B; Supplementary Table S4). All three PARP inhibitors reduced the binding of PARP1 to this matrix in a dose-dependent manner together with its known protein complex partners, such as DNA topoisomerase (TOP1), x-ray repair cross-complementing protein 1 (XRCC5), DNA ligase 3 (LIG3), including PARP2 (Supplementary Fig. S1A and S1B; refs. 40, 41). In addition, olaparib displayed the highest affinity to ferrochelatase and tankyrases (TNKS and TNKS2) including their complex partners like GDP-mannose 4,6-dehydratase (Supplementary Fig. S1A). The latter complexes were also competed by talazoparib (Supplementary Fig. S1A). In contrast, JAK1 was the most potent niraparib-specific interactor. To investigate whether JAK1 enzymatic activity is affected by niraparib, phosphorylation of signal transducer and activator of transcription 5 (STAT5) phosphorylation was measured in stimulated human peripheral blood mononuclear cells. The absence of any modulation of JAK1-induced signaling by niraparib suggested that the interaction of this inhibitor does not affect the catalytic activity of JAK1 (Supplementary Fig. S1C and S1E). Also, oxidoreductases were found to bind niraparib at the high micromolar range, for example, aldehyde dehydrogenase 1 (ALDH1), delta (3,5)-delta(2,4)-dienoyl-CoA isomerase (ECH1), and NmrA-like family domain-containing protein 1 (NMRAL1).

To further evaluate the selectivity profile of niraparib in live cells, we performed a 2D-TPP (22). In addition to the effect on PARP1 discussed above (Fig. 1C; Supplementary Table S2), niraparib induced stabilization of several proteins, including the previously discussed kinase off-targets DYRK1A, CDK16, and deoxycytidine kinase (Fig. 1E; Supplementary Table S5; ref. 42). Interestingly, the only protein other than PARP1 with sub-micromolar affinity for niraparib was LSS (lanosterol synthase/oxidosqualene cyclase, pEC_50_: 6.41; Fig. 1F), a key enzyme in cholesterol biosynthesis pathway that catalyzes cyclization of (S)-2,3 oxidosqualene to lanosterol.

**Figure 2:**
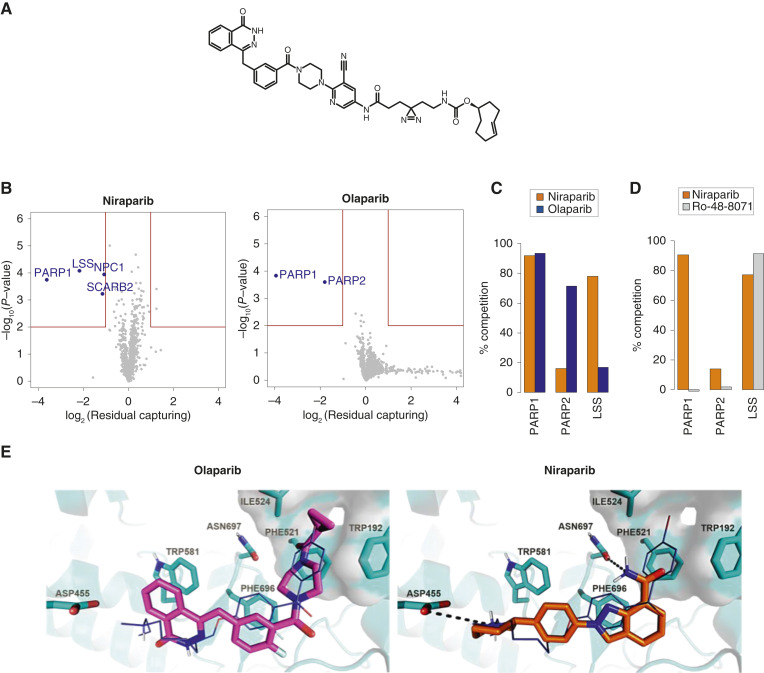
LSS is a novel niraparib-specific off-target. **A,** Chemical structure of the PARP photo affinity probe (TCO-PAL-PARP). **B,** Photoaffinity labeling with the TCO-PAL-PARP probe (1 μmol/L) and competition with olaparib and niraparib (5 μmol/L) in live T47D cells. Volcano plots display all identified proteins. Ratio is calculated as mean (*n* = 3), *P* value is calculated via paired *T* test; significance criteria: *P* < 0.01, |log_2_ fc| > log_2_(1.5), details in Supplementary Table S6. **C,** Bar plots display of mean (*n* = 3) competition for the indicated protein targets in photoaffinity labeling experiments described in **B**. **D,** Photoaffinity labeling experiments with TCO–PAL–PARP probe (1 μmol/L) and competition with niraparib (5 μmol/L) or LSS inhibitor Ro-48-8071 (2 μmol/L). Barplots display mean (*n* = 2) competition for the indicated protein targets. **E,** Docking poses of olaparib (magenta) and niraparib (orange) to human LSS (cyan) overlaid on the co-crystal pose of LSS inhibitor Ro 48-8071 (purple, PDB: 1W6J).

To further examine the observed interaction with LSS, we performed experiments in live cells. A described PARP PAL probe (43) modified here with a trans-cyclooctene group (Fig. 2A, TCO-PAL-PARP probe; ref. 44) was incubated on live cells (43) in the presence of niraparib, which specifically reduced binding to this probe of both LSS and PARP1, but not PARP2 (Fig. 2B and C; Supplementary Table S6). Whereas olaparib, used as a control, did not compete with LSS but instead both PARP1 and PARP2. To investigate the binding mechanism of niraparib to LSS, we performed a cross-competition experiment with the high-affinity LSS inhibitor Ro-48-8071, which binds into the active site of LSS (24). Ro-48-8071 did not affect the binding of the probe to PARP1 or PARP2 but completely competed for LSS from the TCO-PAL-PARP probe (Fig. 2D; Supplementary Table S6). This suggests that both the TCO-PAL-PARP probe and niraparib engage the same binding pocket [i.e., the central active site cavity (24)] of LSS as Ro-48-8071 and that niraparib could potentially inhibit LSS activity. To further validate the direct interaction of niraparib to LSS, we performed differential scanning fluorometry with recombinant full-length LSS, which confirmed the direct binding of niraparib, but not of olaparib, to this enzyme (Supplementary Fig. S1F). Finally, to better understand the molecular reasons for the differential LSS binding of these two PARP inhibitors, we modeled niraparib and olaparib into an existing co-crystal structure of LSS with Ro-48-8071 (Fig. 2E; ref. 24). Both molecules show the same aromatic stacking interaction with Phe696 as the fluorophenyl group in Ro-48-8071. However, like Ro-48-8071, the piperidine ring in niraparib possesses a positively charged nitrogen able to make a key ionic interaction with Asp455, whereas olaparib does not have this functionality. This is expected to be the main driver for the observed difference in LSS binding between niraparib and olaparib, whereas other, smaller clashes (such as the amide carbonyl of olaparib pointing into the hydrophobic pocket between Phe521, Trp192, and Ile524) also contribute to the failure of olaparib to bind to LSS.

In summary, in-depth selectivity profiling demonstrated that niraparib, in contrast to olaparib and talazoparib, is a PARP1-specific PARP inhibitor displaying higher selectivity within the PARP family in both cell lysates and live cells. In addition, using three independent approaches, we identified LSS as a novel niraparib off-target with sub-micromolar potency.

### Niraparib-specific modulation of the cholesterol biosynthesis pathway

**Figure 3:**
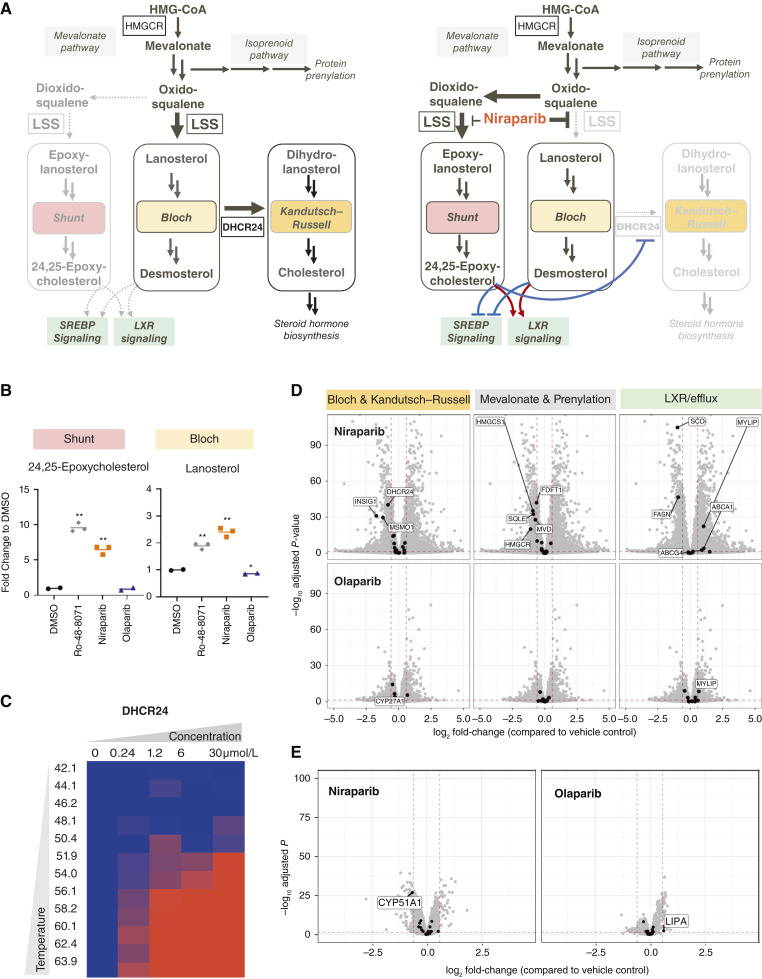
Niraparib-specific modulation of cholesterol biosynthesis pathway. **A,** A scheme of cholesterol biosynthesis pathway, on the right indicated is modulation observed upon partial LSS inhibition (resulting from activation of the 24,25-epoxycholesterol shunt pathway). **B,** Quantification of steroids levels from the cholesterol biosynthesis pathway in breast cancer cell line T47D treated for 48 hours with niraparib (4 μmol/L), olaparib (10 μmol/L) or LSS inhibitor Ro-48-8071 (1 nmol/L). Mean of technical replicates per steroid (*n* = 2–3) is shown (two-tailed unpaired *T* test with Welch’s correction *vs.* DMSO, *, *P* ≤ 0.05, **, *P* ≤ 0.01). Niraparib-specific modulation correlates with partial inhibition of LSS enzyme by Ro-48-8071: increase in 24,25-epoxycholesterol levels pointing out to activation of 24,25-epoxysterol shunt pathway and up-regulation of Bloch pathway metabolites (details in Supplementary Fig. S3C). **C,** Heat map (*n* = 1) displaying relative stabilization (compared with control treatment) of DHCR24 observed upon 48 hours treatment with niraparib (*x*-axis: 0.24, 1.2, 6, and 30 μmol/L) across 12 temperatures (*y*-axis: 42°C–63.9°C) in T47D in 2D-TPP experiment. Olaparib has no significant effect in this cell line on the thermal stability of enzymes in the cholesterol biosynthesis pathway (Supplementary Table S5). **D,** Transcriptomic analysis of T47D cell line treated with niraparib or olaparib (10 μmol/L; 48 hours). Mean fold changes (*n* = 3) and adjusted *P* values from one representative experiment are shown. Black are all detected transcripts belonging to the cholesterol biosynthesis pathway, the significantly [adj. *P* < 0.05, |log_2_ fc| > log_2_ (1.5)] modulated ones are indicated with name. Niraparib-induced modulation leads to the downregulation of several enzymes involved in all branches of the cholesterol biosynthesis pathway (mevalonate, prenylation, Bloch, and Kandutsch–Russell) and to upregulation of genes involved in cholesterol efflux (LXR signaling). The effect size after olaparib treatment is much smaller. **E,** Proteomic analysis of T47D cells treated with niraparib or olaparib (10 μmol/L; 48 hours). Mean fold changes (*n* = 3) and adjusted *P* values from one representative experiment are shown. Black are all detected proteins belonging to the steroid biosynthesis pathway, the significantly [adj. *P* < 0.05, |log_2_ fc| > log_2_ (1.5)] modulated ones are indicated with name. Niraparib decreases CYP51A1 (lanosterol 14α-demethylase) enzyme levels (Supplementary Fig. S4A).

To investigate whether niraparib binding to LSS translates into modulation of its enzymatic activity in live cells, we measured the effects of niraparib on metabolite levels in the cholesterol biosynthesis pathway and compared it with an LSS inhibitor, Ro-48-8071 (24, 45), as well as olaparib. LSS is positioned at a branching point of cholesterol biosynthesis: The Bloch pathway converts lanosterol into desmosterol, which ultimately is reduced to cholesterol by delta (24)-sterol reductase (DHCR24); alternatively, following reduction of any Bloch pathway intermediate by DHCR24, the parallel Kandutsch–Russell (K-R) pathway can be utilized, which also leads to cholesterol production (Fig. 3A; refs. 46, 47). In addition, LSS can also divert metabolic flux through the epoxysterol shunt pathway, which synthesizes 24,25-epoxycholesterol, a key regulator of cellular cholesterol homeostasis (48, 49).

Following the incubation of cancer cell lines with niraparib, olaparib, or Ro-48-8071, the steroid intermediates of these three pathways were analyzed using a dedicated MS-based approach. After 48 hours, a significant increase of a peak containing 24,25-epoxycholesterol (Supplementary Fig. S2A–S2H) was detected with both Ro-48-8071 (10-fold at 1 nmol/L) and niraparib (6.5-fold at 4 μmol/L), whereas no effect was observed with olaparib (Fig. 3B; Supplementary Fig. S3A; Supplementary Table S7). Because the intracellular engagement of olaparib with its primary targets PARP1 and PARP2 could be measured under the same conditions (Supplementary Fig. S3B), an impairment of its cellular penetration can be excluded. This finding suggests that niraparib directly activates the 24,25-epoxysterol shunt pathway in a similar way to an LSS inhibitor and that the observed accumulation of 24,25-epoxycholesterol is not a consequence of altered PARP activity, as suggested by the lack of such regulation by olaparib. In the Bloch pathway, niraparib increased lanosterol levels similarly to the LSS inhibitor Ro-48-8071 (Supplementary Fig. S3B). Other intermediates of this pathway (FF-MAS, 5a-7,24-cholestadiene, and desmosterol/zymosterol) were also elevated upon niraparib treatment, whereas no or minimal accumulation was induced by olaparib (Supplementary Fig. S3C–S3E; Supplementary Table S7). In the K-R pathway, niraparib elicited only mild responses on the levels of intermediates (e.g., downregulation of dihydro-T-MAS, Supplementary Fig. S3C–S3E), suggesting differential modulation of the activities of these two parallel pathways converting lanosterol into cholesterol (46). Notably, reported niraparib-dependent modulation of steroid intermediates in the cholesterol biosynthesis pathway was observed in two different cancer cell lines—breast T47D (Fig. 3B; Supplementary Fig. S3D) and ovarian UWB1.289 (Supplementary Fig. S3E), which strengthens the evidence of a finding beyond a single cell line.

The accumulation of Bloch pathway intermediates might result from the previously reported allosteric inhibition of DHCR24 activity by 24,25-epoxycholesterol, which would hinder the conversion of these intermediates into substrates for the K-R pathway (Supplementary Fig. S3F; ref. 50). As binding of metabolites can lead to thermal stabilization of enzymes, we performed a 2D-TPP analysis of niraparib treated cells and observed thermal stabilization of DHCR24 after 48 hours (Fig. 3C; Supplementary Table S5), consistent with binding of 24,25-epoxycholesterol with DHCR24.

24,25-Epoxycholesterol, lanosterol, and desmosterol regulate cholesterol homeostasis at multiple levels. These include a decrease of cholesterol synthesis and uptake [mediated via the steroid-responsive binding protein (SREBP) pathway by direct interaction with insulin-induced gene 1 (INSIG1)], and induction of cholesterol efflux gene expression [by direct interaction with the nuclear receptor LXR (Supplementary Fig. S3F; refs. 51, 52)]. To investigate if niraparib treatment can lead to modulation of those regulatory feedback loops in cancer cell lines, we performed transcriptomics and proteomics analyses after 48 hours of treatment to enable the accumulation of such downstream effects.

Transcriptomic analysis of cells treated with niraparib revealed significant modulation of cholesterol homeostasis genes following niraparib treatment, whereas only minor effects were detected with olaparib (Fig. 3D; Supplementary Table S8). Specifically, significantly (adjusted *P* < 0.05) downregulated transcripts in the Bloch/K-R pathways were *INSIG1*, *DHCR24*, *MSMO1*, and in the mevalonate and prenylation pathways: *HMGCR*, *HMGCS1*, *SQLE*, *FDFT1*, and* MVD*. These data suggest an activation of the negative feedback loops via INSIG and SREBP. Furthermore, transcript levels of genes involved in cholesterol export, such as *ABCG4*, *ABCA1*, and inducible degrader of the low-density lipoprotein receptor (MYLIP/IDOL), were upregulated, indicating activation of LXR signaling (52). Notably, two genes playing key roles in fatty acid biosynthesis—*SCD* and fatty acid synthase (*FASN*), both known targets of SREBP and LXR signaling—also were downregulated by niraparib but not olaparib.

Proteomic analysis identified niraparib-specific downregulation of CYP51A1 (lanosterol 14-alpha demethylase; Fig. 3E; Supplementary Table S9), an enzyme that converts the LSS products lanosterol and 24,25-hydrolanosterol into FF-MAS and dihydro-FF-MAS, respectively. While CYP51A1 regulation at the RNA level [reported to be regulated via LXR and SREBP signaling (52)] was minor (Supplementary Table S8), changes at the protein level were time-dependent (Supplementary Fig. S4A) and consistent across multiple cell lines (Supplementary Fig. S4B; Supplementary Table S9) analyzed by proteomics, in agreement with previous evidence for a post-translational regulation (47). In addition, in some of those tested cell lines, other enzymes in the cholesterol biosynthesis pathway (HMGCR, HMGCS1, SQLE, and FDFT1) were downregulated (Supplementary Fig. S4B).

In summary, in-depth multi-omics analysis of steroid metabolites, transcripts, and proteins revealed niraparib-specific modulation of the cholesterol biosynthesis pathway, consistent with partial inhibition of LSS and resulting in induction of the 24,25-epoxysterol shunt pathway and the feedback signaling by SREBP and LXR.

### Effect of co-inhibition of PARP and cholesterol biosynthesis pathway on tumor cell viability

**Figure 4:**
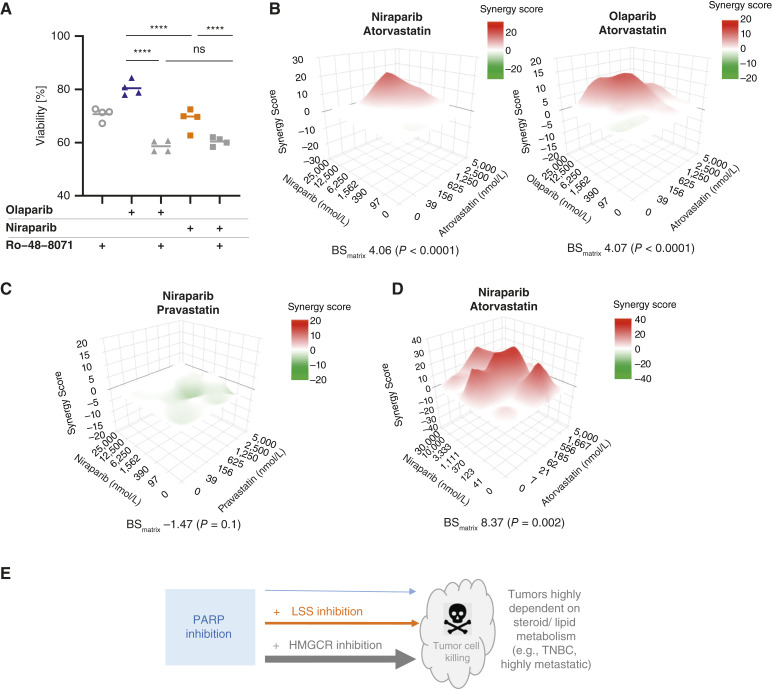
Co-inhibition of PARP and cholesterol biosynthesis pathway improves efficiency in tumor cell killing. **A,** Viability (ATP levels) of HCC70 triple-negative breast cancer (TNBC) cell line after 7-day treatment with niraparib (6.25 μmol/L) and olaparib (6.25 μmol/L) as a single agent or in combination with LSS inhibitor Ro-48-8071 (300 nmol/L). Mean (*n* = 3) for four independent biological experiments is shown (linear mixed-effect model with TukeyHSD contrasts, ****, *P* ≤ 0.0001). Niraparib is more efficient than olaparib in reducing cell viability. Addition of an LSS inhibitor improves both niraparib and especially olaparib efficiency in reducing the viability and makes both inhibitors comparable. **B,** 3-D surface Bliss synergy response for niraparib–atorvastatin and olaparib–atorvastatin combinations after 7-day treatment in MDA-MB-231 TNBC cell line. Red indicates the region of synergy, and green indicates the region of antagonism. Representative of three independent biological experiments (each *n* = 3) is shown as well as the mean BS_matrix_ and *P* value reported (see Supplementary Fig. S5F and S5G for details including other replicates and statistical analysis). Both combinations with lipophilic atorvastatin resulted in a significant synergistic response (significance criteria: mean BS_matrix_ > 3, *P* < 0.05). **C,** 3D surface Bliss synergy response for niraparib–pravastatin, details as in **B**. Combination with hydrophilic pravastatin shows no synergy. **D,** 3D surface Bliss synergy response for niraparib–atorvastatin combination after 7-day treatment in HGS1 (HUB-19-C2-008) patient-derived ovarian cancer organoids (*n* = 4). This combination resulted in significant synergy (see Supplementary Fig. S5H for details). **E,** Model: inhibition of PARP combined with inhibition of cholesterol biosynthesis pathway (e.g., through the niraparib off-target activity on LSS or by combination with LSS inhibitors or more strongly with HMGCR inhibitors such as lipophilic statins) can result in increased cell death of tumors subtypes, which depend on steroid metabolism, like TNBC and/or highly metastatic tumors.

Because aggressive tumor types such as TNBC or brain metastasis (53–57), are characterized by hyperactivation of cholesterol and lipids biosynthesis and LSS inhibitors were shown to impair metastasis dissemination (58–61), we tested the effects of the LSS inhibitor Ro-48-8071 on cell viability in a TNBC, HCC70 cancer cell line, and observed a dose-dependent reduction up to approximately 30% at 300 nmol/L (Supplementary Fig. S5A). In the same cell line, niraparib reduced viability up to 40% at 6.25 μmol/L while olaparib showed only a 20% reduction at the same concentration (Fig. 4A; Supplementary Fig. S5B). Consistent with previous observations, proteomic analysis following incubation with niraparib showed significant downregulation of HMGCR, HMGCS1, SQLE, FDFT1, and CYP51A1, suggesting an induction of the SREBP/LXR signaling, whereas no modulation of these cholesterol biosynthesis enzymes was observed with olaparib (Supplementary Fig. S5C). Because the intracellular engagement of olaparib with its primary target PARP1 could be measured under the same conditions, a reduced cellular penetration of this small molecule can be excluded (Supplementary Fig. S5D). To investigate whether the differential effect on cell viability between niraparib and olaparib could be caused by the inhibitory activity that niraparib exerts on LSS, we tested a combination of Ro-48-8071 with PARP inhibitors. Incubation of Ro-48-8071 together with olaparib resulted in an additive effect of cell killing to levels comparable with those achieved with niraparib alone (Fig. 4A; Supplementary Fig. S5E). The minor increase of cell killing observed when adding Ro-48-8071 to niraparib could be caused by the LSS inhibitor having greater efficiency than niraparib as an LSS inhibitor and 24,25-epoxysterol shunt pathway inducer (Supplementary Fig. S3A). These data suggest that co-inhibition of PARP and LSS can affect cell viability of tumor cells sensitive to modulation of cholesterol metabolism and that the niraparib-specific target-binding profile, which leads to LSS inhibition, could aid this effect.

This observation suggests the possibility that inhibitors acting upstream in the cholesterol biosynthesis pathway would also be able to impact cell viability when combined with PARP inhibitors. Toward this end, potential synergies between PARP inhibitors and statins, which are marketed inhibitors of HMGCR (62), an enzyme catalyzing the rate-limiting step in the mevalonate pathway, were assessed (Fig. 3A; Supplementary Fig. S3F). The effects of combining niraparib or olaparib with either lipophilic (atorvastatin, fluvastatin) or hydrophilic (pravastatin) statins on a matrix of combination ranges were assessed using the Bliss Synergy Score (63). Synergistic effects on the growth of TNBC MDA-MB-231 cells were observed when either niraparib or olaparib were combined with atorvastatin (Fig. 4B; Supplementary Fig. S5G). A similar outcome was observed for combinations with another lipophilic statin, fluvastatin, in a brain metastatic BRCA cell line HCC1954 and the ovarian cancer cell line UWB1.289 (Supplementary Fig. S5F). In each case, synergy was observed at sub-micromolar concentrations of both single agents. Conversely, no effects were observed in combination with pravastatin (Fig. 4C; Supplementary Fig. S5G), a hydrophilic statin that would not permeate cells unless specific transporters from the organic anion-transporting polypeptide family (e.g., OATP1B1) are expressed on the plasma membrane (64). Synergistic effects of the same combinations with lipophilic statins were not observed using the TNBC cell lines MDA-MB-453 and HCC1806 or the BRCA cell line T47D (Supplementary Fig. S5F). Such a distinct response is in line with literature reporting different levels of single-agent sensitivity across cell lines for PARP inhibitors (39) as well as statins (65). Moreover, we could identify some features common to three cell lines that showed synergy in our experiments (in contrast to the cell lines, which did not) based on two comprehensive studies [*n* = 465 tumor samples (66) or *n* = 503 cancer cell lines (54), respectively] proposing a classification of tumors into subtypes. According to those, the cell lines that showed synergy in our manuscript have medium-to-high capability of metastasizing to the brain (54) and belonged to a class named MPS3 (metabolic-pathway-based subtype 3), which shows partial dysregulation for lipid, carbohydrate, and nucleotide metabolism pathways (66). Altogether, our findings highlight a strong dependency of the synergistic response between PARP and statins on specific tumor types and potentially on specific tumor subtypes characterized by differential activation states of the metabolic pathways (66).

To explore the translation of cell line observations to primary tumors, we assessed combinations of niraparib with either atorvastatin or pravastatin in a patient-derived HRp ovarian cancer organoid model (Fig. 4D). Consistent with the findings in the cell lines, synergistic effects on cell viability could be measured with atorvastatin combination, whereas no effects were detected with pravastatin (Fig. 4D; Supplementary Fig. S5H).

In summary, these results indicate that inhibition of PARP combined with modulation of the cholesterol biosynthesis pathway—either affecting LSS activity or more strongly by inhibiting HMGCR with statins—can lead to increased cell death of some tumor types, particularly tumor cells sensitive to modulation of cholesterol metabolism, such as TNBC and/or highly metastatic tumors (Fig. 4E).

## Discussion

When comparing the PARP inhibitors that are approved for oncology indications, olaparib, rucaparib, niraparib, and talazoparib, some differences have been reported at the molecular level, such as efficiency in trapping PARP proteins onto the chromatin, with talazoparib showing a stronger effect than the other three inhibitors (5, 6). The pharmacokinetic profile also distinguishes these molecules, with niraparib displaying the highest volume of distribution (i.e., 1,220 vs. 158 L for olaparib at 300-mg single dose), with the potential of achieving high concentrations in tissues, including brain (67), or olaparib showing the highest maximal plasma concentration (C_max_ equivalent to 13.4 vs. 2.5 μmol/L of niraparib), whereas plasma protein-binding values are comparable (7–10, 68, 69). Different effect on metabolizing enzymes such as the CYP family proteins results in a different spectrum of drug–drug interactions across the PARP inhibitors (70).

Common adverse events such as hematological toxicities (including anemia, neutropenia, or thrombocytopenia; refs. 6, 70, 71) have been reported for the whole class, suggesting that these toxicities might be associated with primary pharmacology. To this end, in addition to PARP trapping (14, 72), also PARP2 has been proposed as a mechanism underlying bone marrow toxicity (73, 74). In line with this hypothesis, PARP1-selective inhibitors are actively being pursued as a novel strategy to improve the therapeutic index of this drug class (75).

Previously, niraparib was reported to have a similar affinity for PARP1 and PARP2 in cell-free assays based on recombinant proteins (36, 76). Our chemoproteomic analysis performed in cell lysates and live cells revealed that in contrast to the other clinical PARP inhibitors olaparib, talazoparib, and rucaparib (69, 75), niraparib displays high selectivity for PARP1 (Fig. 1A; Supplementary Table S1). While niraparib engages with other PARP family members such as PARP2, PARP3, or tankyrases, also linked to potential toxic effects (77, 78), this engagement occurs only at concentrations greater than 10 μmol/L. Within the exposure range measured in clinical settings for niraparib, only one off-target was identified by our molecular profiling, namely LSS, a key enzyme in cholesterol biosynthesis pathway. Although an indication of a weak olaparib interaction with this enzyme was previously reported (43), the in-depth comparison described here for these PARP inhibitors with three independent experimental approaches as well as *in silico* modeling in a co-crystal structure with LSS suggests that this is a niraparib-specific interaction. In addition to the measurement of direct binding of niraparib to LSS, multiomic analysis showed downstream effects consistent with partial inhibition of LSS exclusively by niraparib and not olaparib. Niraparib treatment diverts metabolic flux through the epoxysterol shunt pathway, leading to the accumulation of 24,25-epoxycholesterol, a key regulator of cellular cholesterol homeostasis, which results in the modulation of LXR and SREBP-mediated signaling (48). Although PARP enzymes are reported to regulate several key processes of lipid homeostasis (79), including suppression of SREBP and LXR, the observed modulation of cholesterol homeostasis was induced only by niraparib and not another PARP inhibitor like olaparib, suggesting that it cannot be solely mediated by inhibition of PARP activity. Notably, even though the role of other niraparib off-targets in the cholesterol biosynthesis pathway cannot be excluded at high compound concentrations, PARP1 and LSS are the only two proteins engaged by niraparib at sub-micromolar concentrations. To summarize, the reported in-depth multiomic molecular characterization reveals a distinct poly-pharmacological profile of niraparib not observed with other PARP inhibitors.

LSS has been reported to play a role in oncogenicity, cell self-renewal, and resistance to therapy of BRCA stem cells (58, 59). More importantly, a growing body of evidence suggests a key role of metabolic reprogramming in tumor proliferation, metastasis or resistance, and dependency of such adaptations on specific microenvironmental contexts (57). These could vary across tumor types, subtypes, or within the same patient, for example at different sites or progression stages. For instance, upregulation of cholesterol and lipid biosynthesis has been reported as a hallmark of TNBC, when compared with other breast tumor types (56), and further subcategorization of TNBC has been proposed based on metabolic gene expression, metabolites levels, genomic alterations, and prognoses (66, 80). Moreover, breast cancer-derived brain metastases have been reported to have significant alterations in cholesterol and fatty acid metabolism, adaptations that are proposed to play a key role in the ability of BRCA cells to grow in the brain tissue microenvironment (53–55). Interestingly, multiple lines of evidence suggested that the shift of sterol flux from cholesterol to 24,25-epoxycholesterol synthesis by LSS inhibition in the brain is impeding the progression of glioblastoma (61).

Therefore, we hypothesized that in cell lines highly sensitive to modulation of cholesterol pathways, this poly-pharmacological effect of niraparib in combination with PARP inhibition may lead to increased cell death. Indeed, we demonstrated in the TNBC cell line HCC70, in which niraparib affects cell viability more than olaparib (which does not target LSS), that the combination of olaparib and an LSS inhibitor enhances cell death to levels comparable with niraparib single-agent activity.

While the co-treatment of niraparib with an LSS inhibitor caused a modest increase in cell killing, we demonstrated a synergistic effect when PARP inhibitors were combined with statins. This synergy could be observed at sub-micromolar concentrations with both niraparib and olaparib with lipophilic statins in multiple cell lines reported to have upregulated cholesterol metabolism pathways, highlighting a potential dependency on specific tumor subtypes, for example, characterized by differential activation states of the metabolic pathways, as suggested by other reports (66). As statins inhibit the upstream rate-limiting enzyme of the mevalonate pathway and induce downregulation of overall cholesterol levels as well as intracellular biosynthesis, HMGCR inhibition is expected to overrule niraparib’s ability to inhibit LSS signaling. Therefore, combination of either PARP inhibitor with statins would result in comparable synergistic effects, which is consistent with our observations.

Although several studies have reported an association of statins in combination with anti-cancer therapies (62, 81), the presented findings indicate, to our knowledge for the first time, the potential benefit of combination therapy with statins and PARP inhibitors. As statins are reported to affect cellular pathways beyond cholesterol biosynthesis (82) and the evidence of the impact of statins combinations in oncological indications is still controversial, additional pre-clinical investigations, including animal studies, would be needed to further assess the potential clinical benefit of PARP inhibitors in combination with statins.

## Supplementary Material

Figure S1 Selectivity profiling and Niraparib-LSS interaction

Figure S2 Detection of 24,25-epoxycholesterol

Figure S3 Niraparib specific modulation of cholesterol biosynthesis pathway in cancer cell lines

Figure S4 Niraparib specific modulation of expression levels of proteins involved in cholesterol biosynthesis pathway in cancer cell lines (proteomics)

Figure S5 Co-inhibition of PARP and cholesterol biosynthesis pathway increase efficiency of tumor cell killing

Table S1 panPARP matrix (affinity enrichment chemoproteomics)

Table S2 PARP1 PARP2 engagement (ITDR)

Table S3 Kinobead assay (affinity enrichment chemoproteomics)

Table S4 Niraparib matrix (affinity enrichment chemoproteomics)

Table S5 Selectivity profile live cells (2D-TPP)

Table S6 Photo affinity labelling (PAL)

Table S7 Steroidomics

Table S8 Transcriptomic signature

Table S9 Expression proteomic signature

Supplementary Materials and Methods Supplementary Materials and Methods
