# Supplementary material for: Synergistic Effects of PARP Inhibition and Cholesterol Biosynthesis Pathway Modulation
Source: Cancer Res Commun. 2024 Sep 16;4(9):2427–43. doi: 10.1158/2767-9764.CRC-23-0549 (PMC11403291; doi:10.1158/2767-9764.CRC-23-0549)
Supplement: Figure S1 — Selectivity profiling and Niraparib-LSS interaction [file crc-23-0549_figure_s1_suppsf1.docx]

**Figure S1. Selectivity profiling** **and Niraparib-LSS interaction**


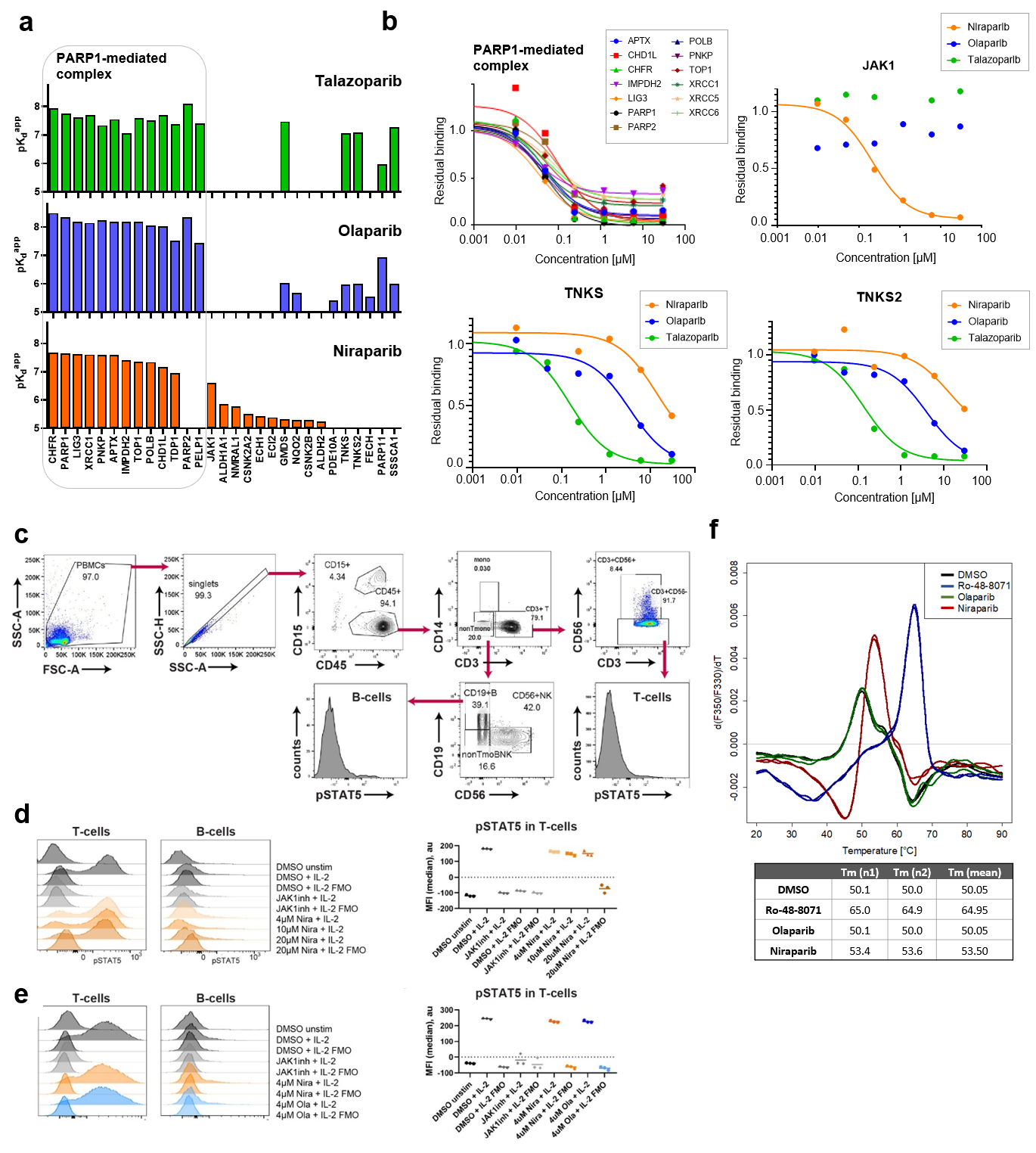


**a,** Proteins identified by affinity enrichment chemoproteomics (AEC) with immobilized niraparib and competition with niraparib, talazoparib or olaparib. Plots depict mean apparent dissociation constants (n=3) for all targets identified across all three experiments and competed from the matrix with 10μM of respective PARP inhibitor. Proteins belonging to PARP1 complex (identified due to protein-protein interactions) are indicated. Details in Supplementary Table 4. **b,** Dose-response curves derived from AEC (a) with immobilized niraparib: PARP1 complex members (example for niraparib) and off-targets JAK1, TNKS and TNKS1. **c,** Gating strategy used for flow cytometry analysis of pSTAT5 levels in T-cells and B-cells to analyze niraparib effect on JAK1 activity. PBMC were pre-treated with indicated compounds for 45 min and stimulated with interleukin (IL)-2 followed by phospho-flow cytometry. **d,** Niraparib titration (4, 10, 20 µM) and comparison to JAK1 inhibitor itacitinib (JAK1 inh, 1 µM): (left) representative pSTAT5 histograms from T- and B-cells and (right) corresponding median fluorescence intensities (MFI) of pSTAT5 staining in T-cells. Fluorescence Minus One (FMO) control samples were stained with all antibodies in the panel minus the pSTAT5-PE antibody. **e,** niraparib vs olaparib comparison (each at 4 µM): (left) representative pSTAT5 histograms from T- and B-cells and (right) corresponding MFI of pSTAT5 staining in T-cells**. f,** Differential scanning fluorometry with recombinant LSS; niraparib (10µM) and LSS inhibitor Ro-48-8071 (10µM) are binding to LSS, whereas olaparib (10µM) not. Tm – melting temperature.
