# Supplementary material for: Synergistic Effects of PARP Inhibition and Cholesterol Biosynthesis Pathway Modulation
Source: Cancer Res Commun. 2024 Sep 16;4(9):2427–43. doi: 10.1158/2767-9764.CRC-23-0549 (PMC11403291; doi:10.1158/2767-9764.CRC-23-0549)
Supplement: Figure S2 — Detection of 24,25-epoxycholesterol [file crc-23-0549_figure_s2_suppsf2.docx]

**Figure S2. Detection of 24,25-epoxycholesterol**


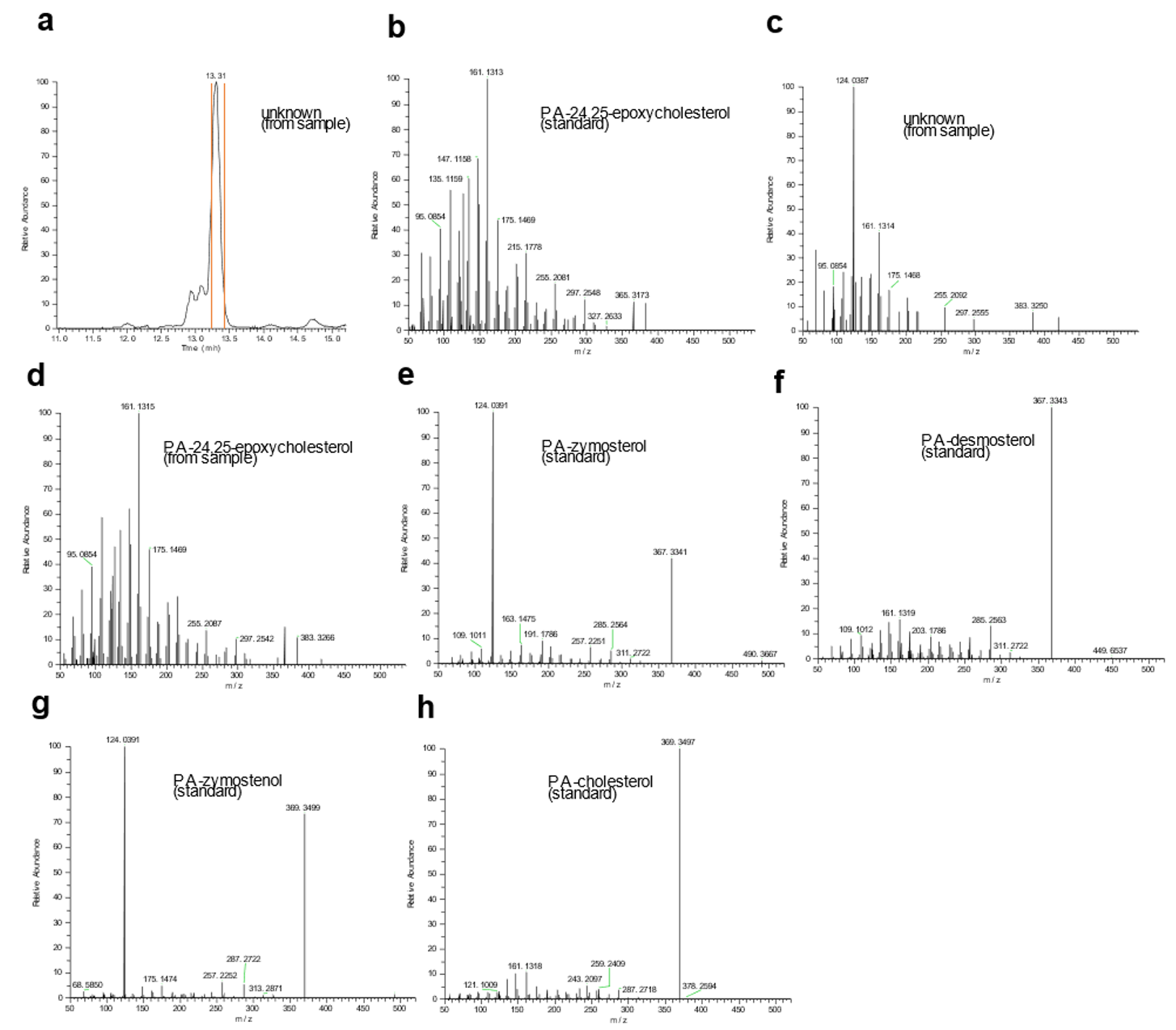


**a**, Extracted ion chromatogram of the peak containing picolinic acid (PA) labelled 24,25-epoxycholesterol. The orange lines indicate the times on which the fragmentation spectra (c) and (d) were taken. **b**, Fragmentation spectrum of PA labelled 24,25-epoxycholesterol standard. **c**, Fragmentation spectrum of an unknown compound isobaric to PA labelled 24,25-epoxycholesterol and with similar fragmentation spectrum to PA labelled 24,25-epoxycholesterol. **d**, Fragmentation spectrum of PA labelled 24,25-epoxycholesterol from a sample. **e**, Fragmentation spectrum of PA labelled Zymosterol standard. **f**, Fragmentation spectrum of PA labelled Desmosterol standard. **g**, Fragmentation spectrum of PA labelled Zymosterol standard. **h**, Fragmentation spectrum of PA labelled Cholesterol standard.

The peak (a) containing PA-24,25-epoxycholesterol (b and d) contains a second compound (c). The structural related compounds PA-Desmosterol (f) and PA-Cholesterol h) also have isobaric compounds that elute slightly later and cannot be baseline separated. These are PA-Zymosterol (e) and PA-Zymosterol (g), respectively. The spectra of PA-Zymosterol (e) and PA-Zymosterol (g) are very similar to their isobaric relatives (f and h) but are dominated by the fragment 124 which results from the PA tag. The fragmentation spectrum of the unknown compound (c) eluting with PA-24,25-epoxycholesterol (d) is also very similar to PA-24,25-epoxycholesterol (b and d) and is dominated by the 124 fragment. Based on the similarities in fragmentation and LC-retention behaviour between the behaviour of PA-Zymosterol (e), PA-Zymosterol (g) and the unknown compound (c) we hypothesise that the unknown compound is PA-24,25-epoxyzymostenol. This hypothesis could not be validated due to the lack of an available standard.
