# Supplementary material for: Synergistic Effects of PARP Inhibition and Cholesterol Biosynthesis Pathway Modulation
Source: Cancer Res Commun. 2024 Sep 16;4(9):2427–43. doi: 10.1158/2767-9764.CRC-23-0549 (PMC11403291; doi:10.1158/2767-9764.CRC-23-0549)
Supplement: Figure S3 — Niraparib specific modulation of cholesterol biosynthesis pathway in cancer cell lines [file crc-23-0549_figure_s3_suppsf3.docx]

**Figure S3. Niraparib specific modulation of cholesterol biosynthesis pathway in cancer cell lines**


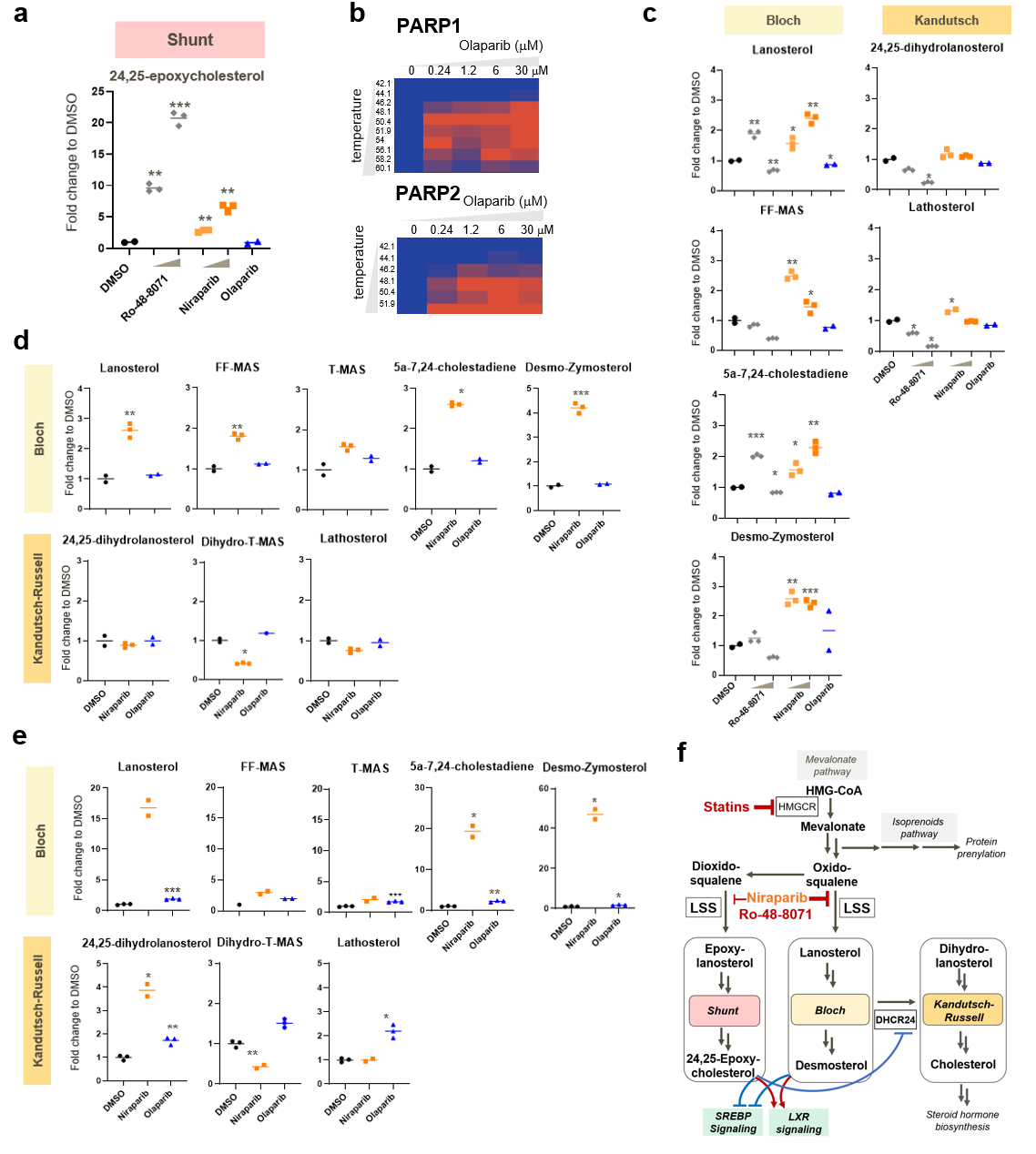


**a**, 24,25-epoxycholesterol levels for additional treatment conditions from the experiment described in Fig. 3b - breast cancer cell line T47D treated for 48h with niraparib (400nM, 4 μM), olaparib (10 μM) or Ro-48-8071 (1 nM, 30 nM). Mean of technical replicates is shown (two-tailed unpaired T-test with Welch’s correction vs. DMSO, * P≤0.05, ** P ≤0.01, *** P ≤0.001 *** P ≤0.001, **** P ≤0.0001); **b**, Heat map displaying relative stabilization (compared to control treatment) of PARP1 and PARP2 observed upon 48h treatment with olaparib (0.24, 1.2, 6, 30 μM) in T47D in 2D-TPP experiment (Supplementary Table 5) **c**, Quantification of other steroids from the cholesterol biosynthesis pathway detected in experiment described in (a) and Fig. 3b. Treatments and statistics as described in (a). Niraparib specific modulation correlates with partial inhibition of LSS enzyme: increased in 24,25-epoxycholesterol levels (activation of Shunt pathway) and up-regulation of some Bloch pathway metabolites levels. **d,** Quantification of steroids from the cholesterol biosynthesis pathway in T47D cell line treated with niraparib or olaparib (10μM; 48h). Mean of technical replicates from one representative experiment is shown (statistics as in (a)). The effect on Kandutsch-Russell metabolites level is minimal (down-regulation of dihydro-T-MAS). Olaparib has no significant effect in this cell line. **e**, Quantification of steroids from the cholesterol biosynthesis pathway in ovarian cancer cell line UWB1.289 treated with niraparib or olaparib (10μM; 48h). Mean of technical replicates from one representative experiment is shown (statistics as in (b)). Niraparib leads to very high (over 15-fold) up-regulation of some Bloch pathway metabolites. The effect on Kandutsch-Russell metabolites level is minimal (up-regulation of 24,25-dihydrolanosterol, down-regulation on dihydro-T-MAS). Olaparib induced modulation is minimal (max. 2-fold). **f**, scheme of the cholesterol biosynthesis pathway, indicated are steps modulated by statins, niraparib and LSS inhibitor Ro-48-8071 treatment and SREBP/INSIG/LXR feedback regulation.
