# Supplementary material for: Synergistic Effects of PARP Inhibition and Cholesterol Biosynthesis Pathway Modulation
Source: Cancer Res Commun. 2024 Sep 16;4(9):2427–43. doi: 10.1158/2767-9764.CRC-23-0549 (PMC11403291; doi:10.1158/2767-9764.CRC-23-0549)
Supplement: Figure S4 — Niraparib specific modulation of expression levels of proteins involved in cholesterol biosynthesis pathway in cancer cell lines (proteomics) [file crc-23-0549_figure_s4_suppsf4.docx]

**Figure S4. Niraparib specific modulation of expression levels of proteins involved in cholesterol biosynthesis pathway in cancer cell lines (proteomics)**


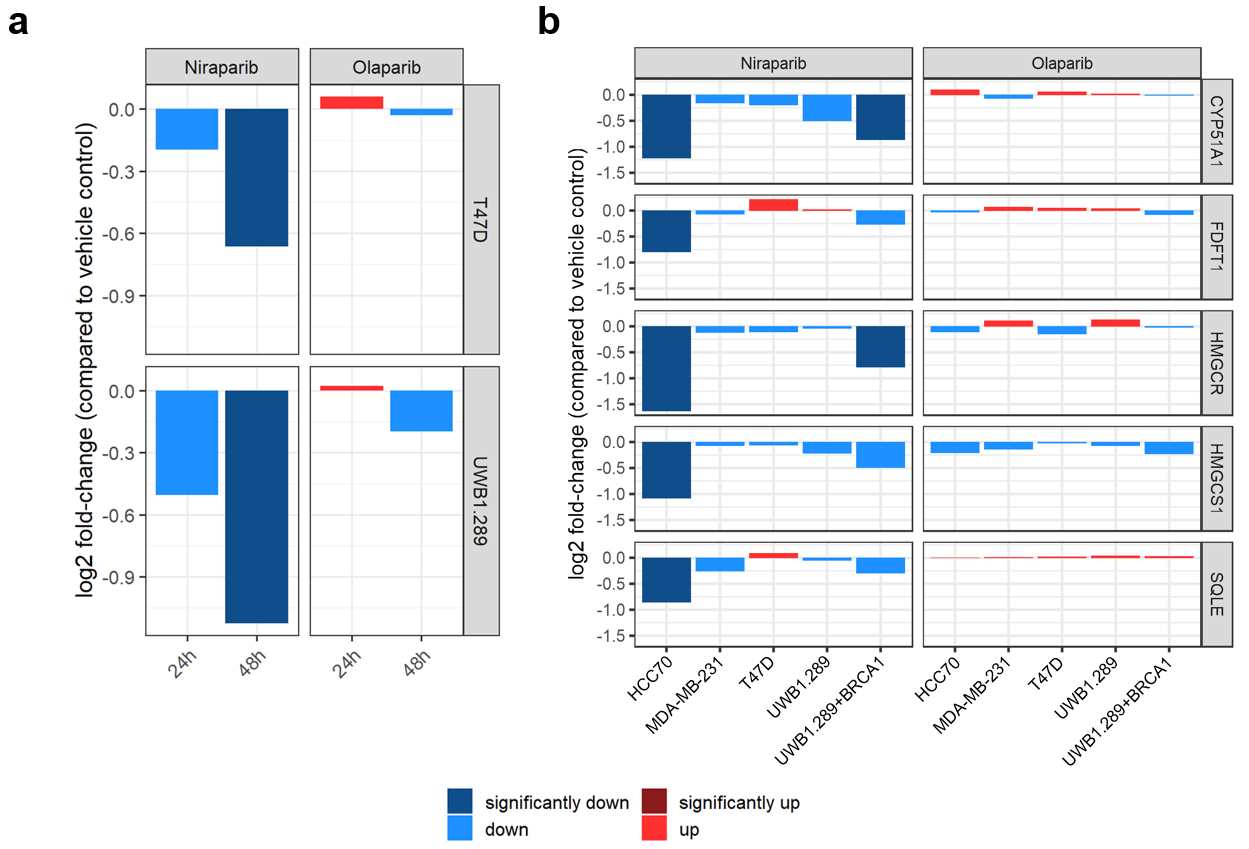


**a**, Changes in CYP51A1 (Lanosterol 14α-demethylase) protein expression levels detected in breast T47D (HRP) and ovarian UWB1.289 (HRD) cells treated with niraparib and olaparib (10 µM; 24 and 48h). Mean (n=3) from one representative experiment is shown, details and statistical analysis in Supplementary Table 9. The significant (adj. p < 0.05, |log2 fc| > log2(1.5)) modulation is indicated with dark blue or dark red color. Niraparib-specific down-regulation of CYP51A1 in those 2 cell lines is time depended and become significant first after 48h treatment. **b**, Changes in protein expression levels of proteins belonging to the cholesterol biosynthesis pathway identified after 24h treatment with niraparib and olaparib (10 µM) across tested cell lines. Five significantly (adj. p < 0.05, |log2 fc| > log2(1.5)) modulated enzymes has been identified in HCC70 cells: CYP51A1, HMGCS1, HMGCR, FDFT1, SQLE. Details and statistical analysis in Supplementary Table 9.
