# Supplementary material for: Synergistic Effects of PARP Inhibition and Cholesterol Biosynthesis Pathway Modulation
Source: Cancer Res Commun. 2024 Sep 16;4(9):2427–43. doi: 10.1158/2767-9764.CRC-23-0549 (PMC11403291; doi:10.1158/2767-9764.CRC-23-0549)
Supplement: Figure S5 — Co-inhibition of PARP and cholesterol biosynthesis pathway increase efficiency of tumor cell killing [file crc-23-0549_figure_s5_suppsf5.docx]

**Figure S5: Co-inhibition of PARP and cholesterol biosynthesis pathway increase efficiency of tumor cell killing.**


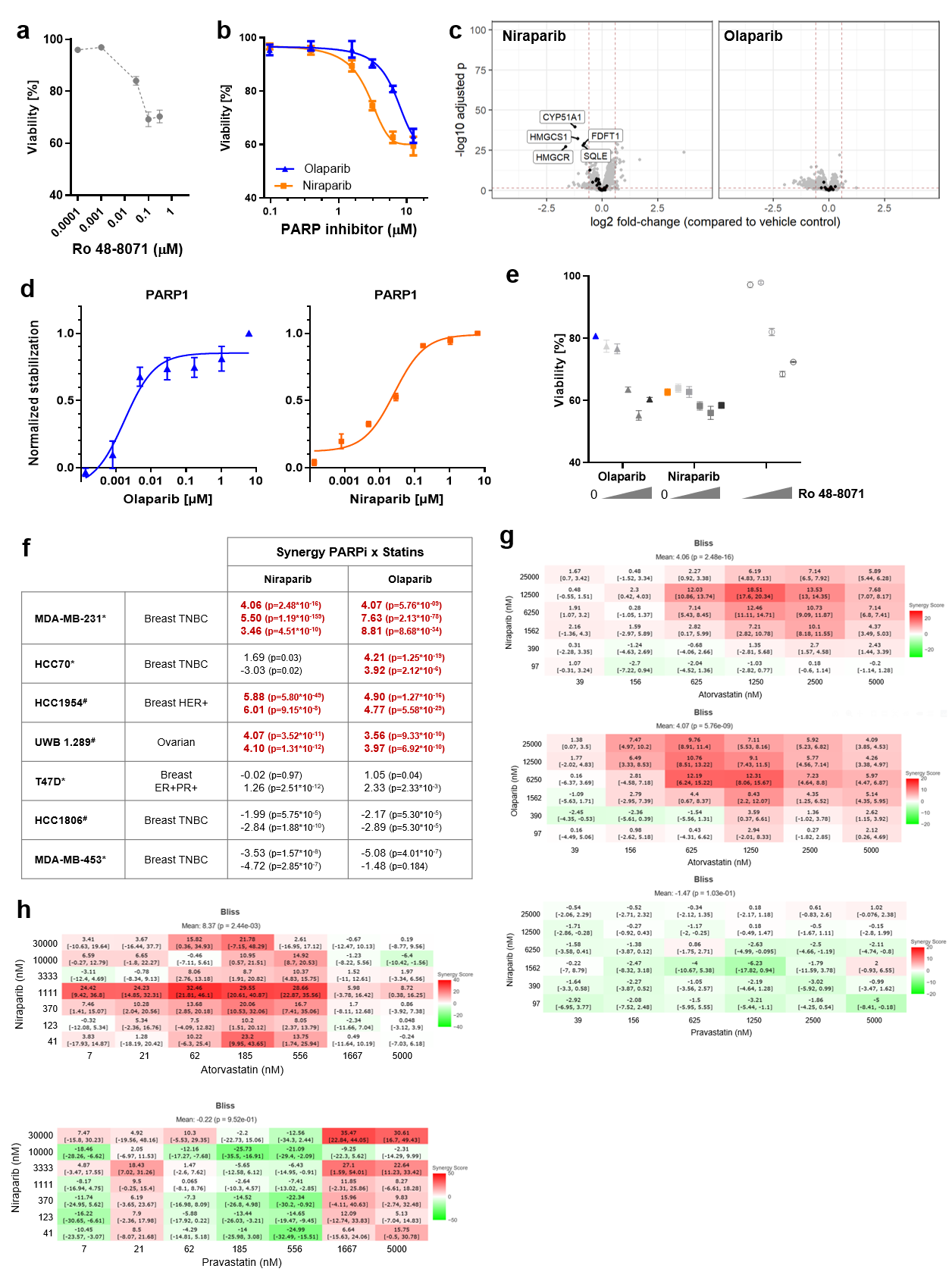


**a,** Viability (ATP levels) of HCC70 triple negative breast cancer (TNBC) cell line after 7-days treatment with concentration range (0.1 – 300 nM) of LSS inhibitor Ro-48-8071. Mean with SEM (n=8) is shown for one representative experiment. **b**, Viability (ATP levels) of HCC70 cell line after 7-days treatment with concentration range of niraparib or olaparib (0.097-12.5 μM). Mean with SEM (n=4) is shown for one representative experiment. At low micromolar concentration niraparib is more potent than olaparib **c**, Proteomic analysis of HCC70 cells treated with niraparib and olaparib (10μM; 48h). Mean (n=3) from one representative experiment is shown. Details and statistical analysis in Supplementary Table 9. Black are all detected proteins belonging to the steroid biosynthesis pathway, the significantly (adj. p < 0.05, |log2 fc| > log2(1.5)) modulated ones are indicated with name. Niraparib down-regulates expression of 5 enzymes involved in the cholesterol biosynthesis: CYP51A1 (Lanosterol 14α-demethylase), HMGCS1, HMGCR, FDFT1, SQLE. **d**, Dose-response curve (at 48°C) of thermal stabilization of PARP1 in HCC70 after treatment with olaparib or niraparib. Mean with SEM (n=4 or n=3, respectively) is shown. Both compounds are permeant and engage PARP1 with similar potency. **e**, Viability (ATP levels) of HCC70 cells after 7-days treatment with niraparib (6.25μM) and olaparib (6.25μM) as single agent or in combination with LSS inhibitor Ro-48-8071 (0.1nM – 300nM). Mean with SEM (n=3) from one representative experiment is shown. Addition of LSS inhibitor improves both niraparib and olaparib efficiency in reducing viability and makes both inhibitors comparable. The effect size on olaparib is more prominent. **f**, Synergy response for niraparib or olaparib combinations with lipophilic statins (*atorvastatin, ^#^fluvastatin) assessed by the Bliss Synergy Score (BS) on a matrix of concentration after 7-days treatment in indicated cell lines. Mean BS_matrix_ and P value for at least two independent biological replicates (each in at least n=3) is reported. Synergy for a given combination was classified as significant (values in red) when the mean BS_matrix_ was > 3, P value < 0.05. **g,** Bliss synergy response (corresponding to the 3D plots in Fig. 4b and c) for niraparib–atorvastatin, olaparib-atorvastatin and niraparib-pravastatin combination  after 7-days treatment in MDA-MB-231 TNBC cell line. Representative of 3 independent biological experiments (each n=3) is shown. Heat-maps indicates BS and 95% CI for each combination mix. Red indicates synergy (BS>0), green antagonism (BS<0). Reported are also mean Bliss Score for the matrix (BS_matrix_) and P value. Synergy for a given combination was classified as significant when the mean BS_matrix_ was > 3, P value < 0.05, synergy was detected in one region covering at least two neighboring concentrations of each compound with BS>3 and positive 95% CI. Only combinations with lipophilic atorvastatin resulted in a significant synergistic response. **h,** Bliss synergy response for niraparib–atorvastatin combination (corresponding to the 3D plot in Fig. 4d) as well as niraparib-pravastatin after 7-days treatment in HUB-19-C2-008 patient-derived ovarian cancer organoids (n=4). Annotations as described in (g). Only combination niraparib-atorvastatin resulted in a significant synergistic response.
