# Supplementary material for: Synergistic Effects of PARP Inhibition and Cholesterol Biosynthesis Pathway Modulation
Source: Cancer Res Commun. 2024 Sep 16;4(9):2427–43. doi: 10.1158/2767-9764.CRC-23-0549 (PMC11403291; doi:10.1158/2767-9764.CRC-23-0549)
Supplement: Supplementary Materials and Methods [file crc-23-0549_supplementary_materials_and_methods_suppsm.pdf]

Supplementary Material and Methods

Chemical synthesis

General procedures

Reagents were purchased from Sigma Aldrich (Germany), Enamine (Ukraine), and abcr (Germany) and used without further purification. All solvents, including anhydrous solvents, were used as obtained from the commercial sources. Air and water-sensitive reagents and reactions were generally handled under argon atmosphere. The reaction progress was monitored by thin layer chromatography (TLC) on Merck silica gel plates 60 F254. Detection was executed with a UV-cabinet HP-UVIS (Biostep) at 254 nm or with potassium permanganate staining. Flash chromatographic purification was performed on a Biotage Isolera One purification system using KP-Sil or SNAP Ultra C18 flash cartridges. Nuclear magnetic resonance (NMR) spectra were recorded on a Bruker Avance (400 MHz) NMR system at 298 K. Chemical shifts (δ) are given in parts per million (ppm), coupling constants (J) given in hertz (Hz), and multiplicity reported using standard abbreviations. Only signals for the main conformers are reported. Ultra-high performance liquid chromatography (UHPLC)/MS analyses were performed on Agilent 1290 series equipment consisting of an Agilent 1290 quaternary pump, a 1290 sampler, a 1290 thermostatted column compartment, and a 1290 diode array detector VL+ equipped with a quadrupole liquid chromatography (LC)/MS 6120 and an Infinity 1260 Evaporative Light Scattering Detector (ELSD). The analytical column used was an Agilent Eclipse Plus Phenyl-Hexyl (2.1 x 50 mm, 3.5 µm) operated at 40 °C and 1.0 ml/min with a gradient (5% B for 0.2 min, 5% to 100% B in 3.3 min, 100% B for 1.0 min) using water and acetonitrile, both containing 0.1% trifluoroacetic acid (TFA) as solvents. Compound purity was determined by ELSD monitoring. Tert-butyl 4-(5-amino-3-cyanopyridin-2-yl)piperazine-1-carboxylate and 3-((4-Oxo-3,4-dihydrophthalazin-1-yl)methyl) benzoic acid were synthesized according to literature-described procedures[1,2].

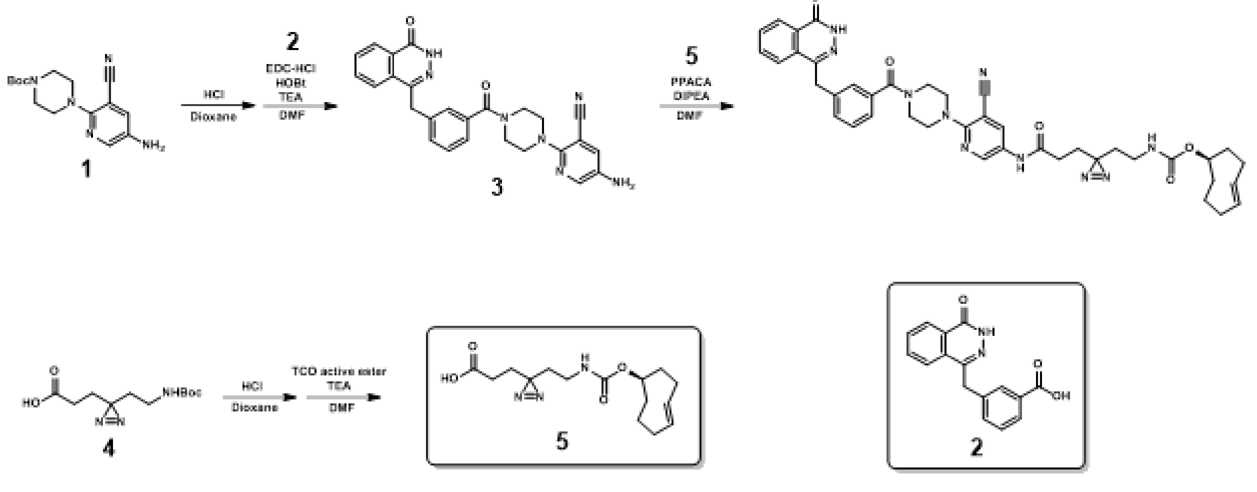

Compound **1** was synthesized according to literature procedure[1] starting from 2-(piperazin-1-yl)nicotinonitrile. Boc deprotection of **1** and subsequent amide coupling with literature-described acid **2**[2] provided amine **3**. Boc deprotection of diazirine molecule **4** followed by reaction with *(E)*-cyclooct-4-en p-NPE active ester yielded TCO precursor **5**, which resulted in TCO probe **6** after amide formation with compound **3**.

Synthesis of (3)

5-Amino-2-(4-(3-((4-oxo-3,4-dihydrophthalazin-1-yl)methyl)benzoyl)piperazin-1-yl)nicotinonitrile

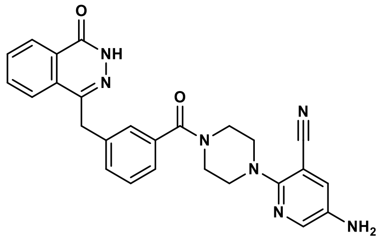

Amine **1** (303 mg, 1.00 mmol, 1.0 eq) was dissolved in dioxane (3.75 ml), and a 4 M solution of HCl in dioxane (1.25 ml, 5.00 mmol, 5.0 eq) was added. The reaction mixture was stirred at room temperature overnight. The mixture was evaporated to dryness and the residue was dissolved in Dimethylformamide (DMF; 10 ml). Acid **2** (280 mg, 1.00 mmol, 1.0 eq) and hydroxybenzotriazole (HOBt; 162 mg, 1.20 mmol, 1.20 eq) were added, and the mixture was cooled to 0 °C. 1-ethyl-3-(3-dimethylaminopropyl)carbodiimide hydrochloride (EDC-HCl; 230 mg, 1.20 mmol, 1.2 eq) and triethylamine (TEA) were added, and the reaction mixture was warmed up to room temperature overnight. The solvent was removed under reduced pressure and the residue was resuspended in saturated aqueous sodium bicarbonate and extracted with dichloromethane (DCM). The organic phase was washed with brine, dried over magnesium sulphate, filtered, and evaporated under reduced pressure. The crude product was purified by column chromatography using a linear gradient of DCM and methanol to obtain 272 mg (0.58 mmol/58%) of the title compound.

^{1}H NMR (DMSO-*d*$_6$): δ 12.61 (s, 1H), 8.25 (dd, J = 7.8, 1.0 Hz, 1H), 7.98 (d, J = 8.2 Hz, 1H), 7.91 (d, J = 2.9 Hz, 1H), 7.89–7.85 (m, 1H), 7.84–7.78 (m, 1H), 7.41–7.34 (m, 3H), 7.28–7.24 (m, 2H), 5.35 (s, 2H), 4.36 (s, 2H), 3.73 (bs, 2H), 3.39 (bs, 2H), 3.21 (bs, 2H), 3.08 (bs, 2H); **UHPLC/MS**: (*m/z*): 466 $(M + H)^+$.

Synthesis of (5)

(R,E)-3-(3-(2-(((Cyclooct-4-en-1-yloxy)carbonyl)amino)ethyl)-3H-diazirin-3-yl)propanoic acid

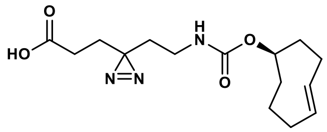

Diazirine **4** (448 mg, 1.74 mmol, 1.0 eq) was dissolved in dioxane (7.0 ml), and a 4 M solution of HCl in dioxane (8.7 ml, 34.8 mmol, 20 eq) was added. The reaction mixture was stirred at room temperature, and the end of the reaction was monitored by TLC and NMR. After evaporation to dryness the residue was dissolved in DMF (17 ml), TEA (0.72 ml, 5.22 mmol, 3.0 eq) and TCO p-NPE active ester (507 mg, 1.74 mmol, 1.0 eq) were added, and the reaction mixture was stirred at room temperature overnight. The solvent was removed under reduced pressure and the residue was suspended in ethyl ethanoate (EtOAc). It was then washed with 0.5 M HCl and extracted with EtOAc twice. The combined organics were dried over magnesium sulphate, filtered, and evaporated. The crude product was purified by column chromatography using a linear gradient of DCM and methanol to obtain 432 mg (1.40 mmol/80%) of the title compound.

^{1}H NMR (CD_3OD): δ 5.69 (ddd, J = 14.9, 11.0, 3.5 Hz, 1H), 5.54 (ddd, J = 15.7, 11.0, 3.1 Hz, 1H), 4.82 (dd, J = 10.3, 5.2 Hz, 1H), 3.05– .98 (m, 2H), 2.44–2.32 (m, 1H), 2.30–2.19 (m, 3H), 2.11 (t, J = 7.6 Hz, 2H), 2.11–2.03 (m, 1H), 1.92–1.80 (m, 1H), 1.76 (t, J = 7.6 Hz, 2H), 1.77–1.71 (m,

1H), 1.71–1.60 (m, 2H), 1.57 (t, J = 7.0 Hz, 2H), 1.31–1.22 (m, 1H); **^{13}C NMR; CD_3OD):** δ 136.4, 132.5, 71.4, 42.0, 36.7, 35.2, 34.1, 33.7, 30.8, 29.0, 29.0; **UHPLC/MS**: (*m/z*): 619 $(2M + H)^+$.

Synthesis of TCO-PAL-PARP probe (6)

(R,E)-cyclooct-4-en-1-yl (2-(3-(3-((5-cyano-6-(4-(3-((4-oxo-3,4-dihydrophthalazin-1-yl)methyl) benzoyl)piperazin-1-yl)pyridin-3-yl)amino)-3-oxopropyl)-3H-diazirin-3-yl)ethyl)carbamate

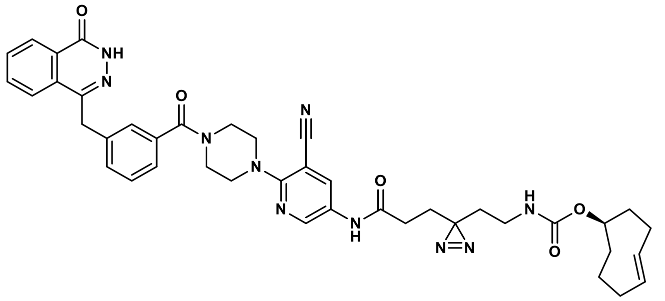

To a mixture of compounds **3** (71 mg, 0.15 mmol, 1.0 eq) and **5** (47 mg, 0.15 mmol, 1.0 eq) in DMF (1.5 ml), N,N-Diisopropylethylamine (DIPEA; 80 µL, 0.46 ml, 3.0 eq) and propylphosphonic acid cyclic anhydride (50% [v/v] in EtOAc, 90 µL, 0.30 mmol, 2.0 eq) were added, and the reaction mixture was stirred at room temperature for 2 hours. The mixture was diluted with EtOAc, washed with saturated aqueous sodium bicarbonate and 5% aqueous lithium chloride. The organic phase was washed with brine, dried over magnesium sulphate, filtered, and evaporated under reduced pressure. The crude product was purified by reverse phase column chromatography using a linear gradient of water and methanol to obtain 19 mg (0.03 mmol/17%) of the analytically pure title compound.

^{1}H NMR (DMSO-d_6): δ 12.61 (s, 1H), 10.23 (s, 1H), 8.50 (d, J = 2.5 Hz, 1H), 8.29 (d, J = 2.5 Hz, 1H), 8.25 (dd, J = 7.8, 1.0 Hz, 1H), 7.97 (d, J = 8.1 Hz, 1H), 7.91–7.86 (m, 1H), 7.85–7.79 (m, 1H), 7.44–7.35 (m, 3H), 7.30–7.25 (m, 1H), 7.12 (t, J = 5.5 Hz, 1H), 5.63 (ddd, J = 14.6, 11.0, 3.4 Hz, 1H), 5.50 (ddd, J = 15.5, 11.0, 2.9 Hz, 1H), 4.71 (dd, J = 10.1, 4.9 Hz, 1H), 4.36 (s, 2H), 3.73 (bs, 2H), 3.55 (bs, 2H), 3.41 (bs, 4H), 2.88 (q, J = 6.9 Hz, 2H), 2.35–2.21 (m, 1H), 2.20–2.05 (m, 5H), 2.05–1.95 (m, 1H), 1.84–1.77 (m, 1H), 1.75 (t, J = 7.5 Hz, 2H), 1.71–1.57 (m, 2H), 1.52 (t, J = 7.1 Hz, 2H), 1.49–1.38 (m, 1H), 1.20–1.11 (m, 1H); **^{13}C NMR (DMSO-d_6):** δ 170.2, 169.0, 159.4, 157.0, 155.6, 145.0, 143.4, 138.5, 135.8, 135.0, 133.7, 133.5, 131.6, 131.5, 129.9, 129.1, 128.7, 128.6, 127.9, 127.3, 126.1, 125.6, 125.3, 117.1, 95.4, 68.5, 40.4, 37.3, 35.3, 33.9, 32.5, 32.2, 30.2, 29.6, 27.6, 27.4, 27.4; **UHPLC/MS**: (*m/z*): 757 $(M + H)^+$, t_R = 3.03 min (purity: >99.9 %).

References

1. Phthalazinone derivatives Patent WO2002036576A1. (2010).
2. Howard, R.T., *et al.* Structure-Guided Design and In-Cell Target Profiling of a Cell-Active Target Engagement Probe for PARP Inhibitors. *ACS Chem Biol* **15**, 325-333 (2020).
